# Recent Advances in Polymer Electrolyte Membrane Water Electrolyzer Stack Development Studies: A Review

**DOI:** 10.1021/acsomega.4c10147

**Published:** 2025-03-04

**Authors:** Murat Kıstı, Bulut Hüner, Abdelmola Albadwi, Emre Özdoğan, İlayda Nur Uzgören, Süleyman Uysal, Marise Conağası, Yakup Ogün Süzen, Nesrin Demir, Mehmet Fatih Kaya

**Affiliations:** a Erciyes University, Energy Systems Engineering Department, Heat Engineering Division, 38039 Kayseri, Türkiye; b Osmaniye Korkut Ata University, Directorate of Research and Innovation, 80000 Osmaniye, Türkiye; c Erciyes University, Mechanical Engineering Department, 38039 Kayseri, Türkiye; d Erciyes University, Electrical and Electronics Engineering Department, 38039 Kayseri, Türkiye; e Erciyes University, Graduate School of Natural and Applied Sciences, Energy Systems Engineering Department, 38039 Kayseri, Türkiye; f Erciyes University H2FC Hydrogen Energy Research Group, 38039 Kayseri, Türkiye; g Erciyes University, ArGePark Research Building, 38039 Kayseri, Türkiye; h Bataryasan Enerji San. ve Tic. A.Ş., Erciyes Teknopark, Yıldırım Beyazıt Mah., Aşık Veysel Bul., No: 63/B, 38039 Melikgazi/Kayseri, Türkiye

## Abstract

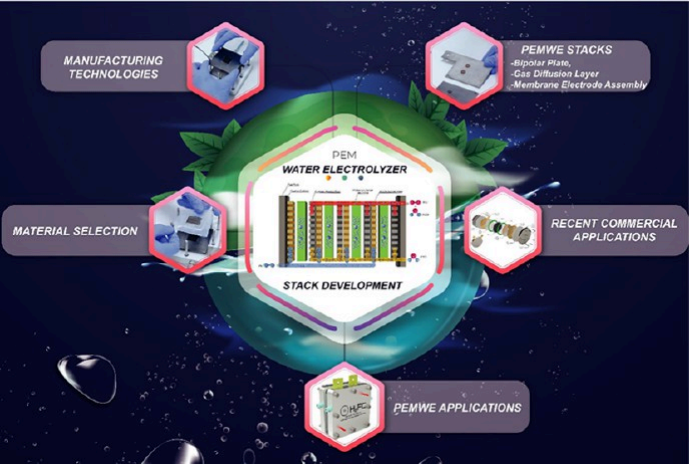

Polymer electrolyte membrane water electrolyzers have significant advantages over other electrolyzers, such as compact design, high efficiency, low gas permeability, fast response, high-pressure operation (up to 200 bar), low operating temperature (20–80 °C), lower power consumption, and high current density. Moreover, polymer electrolyte membrane water electrolyzers are a promising technology for sustainable hydrogen production due to their easy adaptability to renewable energy sources. However, the cost of expensive electrocatalysts and other construction equipment must be reduced for the widespread usage of polymer electrolyte membrane water electrolyzer technology. In this review, recent improvements made in developing the polymer electrolyte membrane water electrolyzer stack are summarized. First, we present a brief overview of the working principle of polymer electrolyte membrane water electrolyzers. Then, we discuss the components of polymer electrolyte membrane water electrolyzers (base materials such as membranes, gas diffusion layers, electrocatalysts, and bipolar plates) and their particular functions. We also provide an overview of polymer electrolyte membrane water electrolyzer’s material technology, production technology, and commercialization issues. We finally present recent advancements of polymer electrolyte membrane water electrolyzer stack developments and their recent developments under different operating conditions.

## 1 Introduction

Due to rapid population growth and the development of innovative technologies in developed and developing countries, global energy consumption is increasing daily. Today, fossil-based fuels (such as coal, oil, and natural gas) are still needed to meet most of the energy needs. When fossil-based fuels are burned, they release polluting gases into the atmosphere, resulting in greenhouse gas emissions (CO_*x*_, SO_*x*_, NO_*x*_) and air pollution. The increased air pollution worldwide may result in climate changes, increasing the greenhouse effect, and global warming. Therefore, researchers continue searching for sustainable, environmentally friendly, and clean energy sources that reduce environmental pollution. These clean energy sources are renewable or alternative (wind, solar, etc.), and optimal utilization minimizes environmental effects. The global renewable generation capacity is estimated to be 2799 GW at the end of 2020. The largest share (around 43%) of the installed power is covered by hydroelectric energy with a capacity of 1211 GW. Wind energy (733 GW) and solar energy (714 GW) account for 26.19% and 25.5% of the total installed capacity, respectively.^1^ However, the worldwide revenue from renewable energy sources may exceed $23 billion by 2026, and energy storage requirements will be estimated to nearly triple current values by 2030.^2^ In times of excessive power consumption, as indicated in the Duck Curve, generating electricity using fossil fuels is a possible solution, especially since photovoltaic energy is not included in the grid. However, this situation conflicts with the carbon-emission-lowering targets of the countries. So, it is seen as a better solution to store the produced energy when the energy production is high and it is consumed too much. At the same time, hydrogen energy storage systems are considered an alternative energy carrier.^3^ Hydrogen can be stored physically in both forms, gas and liquid. Moreover, hydrogen storage at high pressure is a standard technology with a gravimetric density higher than those of other storage methods. However, there are safety precautions during hydrogen storage and transport and challenges in obtaining industrially suitable materials for hydrogen storage.^4^ Hydrogen has been considered an energy carrier in future society due to its zero-emission, environmental cleanliness, and high energy density. Using water electrolysis as a source of hydrogen production offers an opportunity to reduce energy costs and make renewable energy storable and portable. Currently, water electrolysis technologies for hydrogen production are divided into three main classes according to the type of electrolyte and operating conditions: polymer electrolyte membrane water electrolysis (PEMWE), alkaline water electrolysis (AWE), and solid oxide electrolysis (SOEC). Among these methods, PEMWEs are superior to other electrolysis systems because they have high efficiency, high-purity hydrogen production, low gas permeability, fast response, high-pressure operation, low operating temperature, lower power consumption, and higher current density.^5−7^ Considering all of these advantages, PEMWEs are the most suitable technology for working with alternative energy sources, balancing grid loads with dynamic and intermittent. It is also thought to meet electrical fluctuations thanks to its fast commissioning feature.^8^ Although there are many studies in the literature on various aspects of PEMWE, relatively few studies discuss cell and stack development. Therefore, the main aim of this study is to consider the improvements of PEMWE cells and stacks for green hydrogen production and their recent developments under different operating conditions. Furthermore, this review focuses on the PEMWE cell and its key components (such as bipolar plate (BP), gas diffusion layer (GDL), and membranes), PEMWE stacks and systems, applications, and manufacturing technologies of PEMWEs. Finally, the stages of commercialization of PEMWEs are discussed, and recommendations for future research design and development of PEMWEs are presented. This Perspective stands out by providing a comprehensive analysis of recent advancements in the development of PEMWEs, specifically focusing on stack development under diverse operational conditions. Unlike prior studies that primarily emphasize isolated aspects of PEMWE technology, this work integrates discussions of material innovations, manufacturing processes, and component optimization within the context of stack performance. By synthesizing findings from diverse studies, it offers a holistic perspective on how individual components interact to influence the overall system efficiency. Moreover, this review addresses cost-reduction strategies for PEMWE systems, an area that remains underexplored in the existing literature, by delving into advanced material technologies and novel manufacturing techniques such as additive manufacturing. This unique approach not only underscores technological advancements but also bridges gaps in knowledge, paving the way for scalable industrial applications of PEMWE stacks.

## 2 PEMWE Cell and Stacks

**Figure 1:**
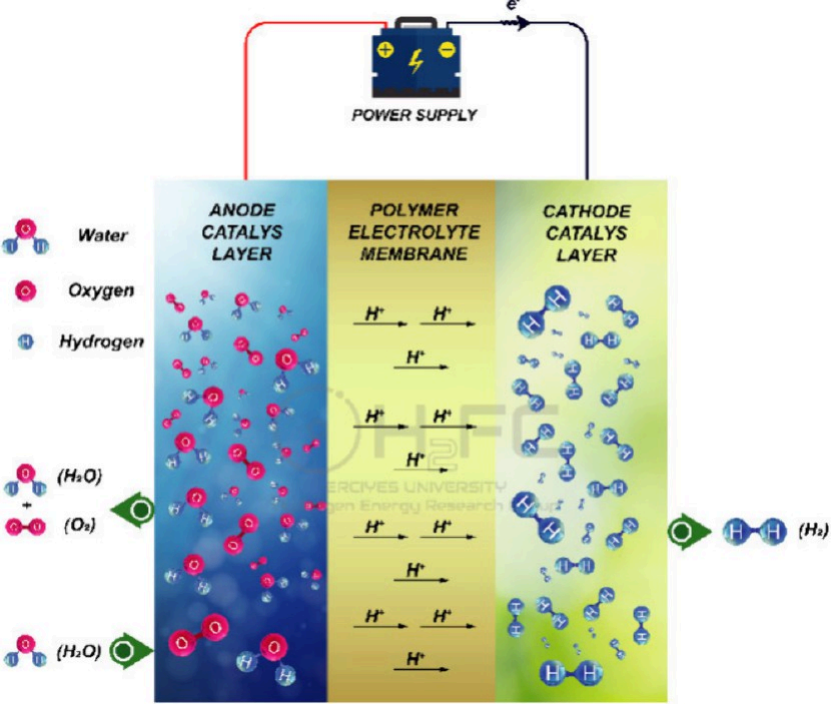
A schematic representation of single-cell PEMWE.

Grubb discovered the first PEMWE in the early fifties, and General Electric (GE) developed it based on the Solid Polymer Electrolyte (SPE) concept to overcome the drawback of AWEs.^9,10^ PEMWE technology works similarly to PEM fuel cell technology where solid perfluoro sulfonic acid (PFSA) based membranes such as Nafion, Flemion, Aciplex, Fumapem, etc. are utilized in electrolytes. Moreover, catalysts deposited on the membrane surface can move protons from the reaction sites to the electrolyte, which can help to significantly reduce the mass transport limitation. Therefore, a robust electrolyte solution is unnecessary to improve ionic conductivity in PEMWEs, and only pure water is supplied to the electrolyzer.^11^ In PEMWE, water is electrochemically separated into oxygen (anode electrode) and hydrogen (cathode electrode) gas with the help of a DC current. The PEMWE process consists of two electrochemical half-reactions: the oxygen evolution reaction (OER) at the anode electrode and the hydrogen evolution reaction (HER) at the cathode electrode. The electrochemical reactions at the anode and cathode sides are given as eqs 1 and 2:^12^anode side
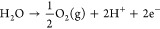
1cathode side
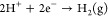
2In PEMWE, water is pumped to the anode side, and it is separated into protons (H^+^), oxygen (O_2_), and electrons (e^–^). Subsequently, these protons pass through a membrane known as an SPE and travel to the cathode side. The oxygen separated from the water is removed from the cell by the anode side and unreacted water. The electrons move from the anode side to the cathode side via an external circuit, and then the protons and electrons recombine to generate hydrogen gas.^13^ The working principle of single-cell PEMWE can be seen in Figure 1.

**Figure 2:**
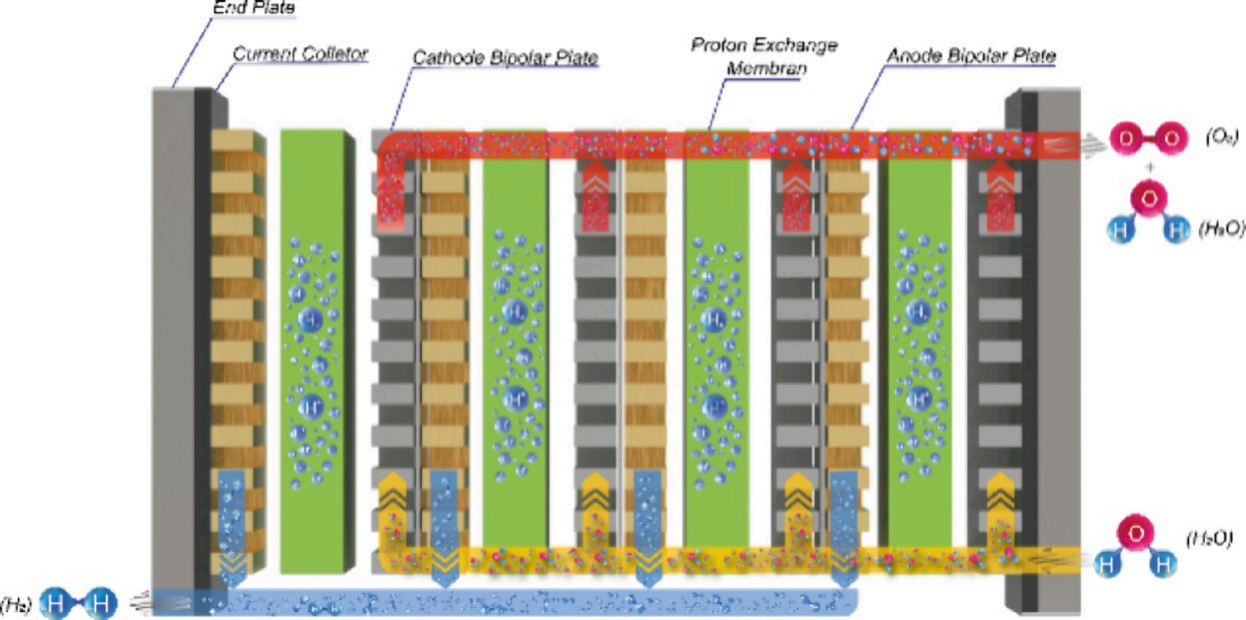
Schematic view of a PEMWE stack.

The hydrogen production rate of a single-cell PEMWE is limited by the active surface area of the membrane electrode assembly (MEA). Therefore, single-cell PEMWE can be connected in series to achieve a higher hydrogen production rate with a higher purity. Figure 2 shows a schematic view of a PEMWE stack in which cells are combined in series to produce sufficient power for industrial applications.

**Figure 3:**
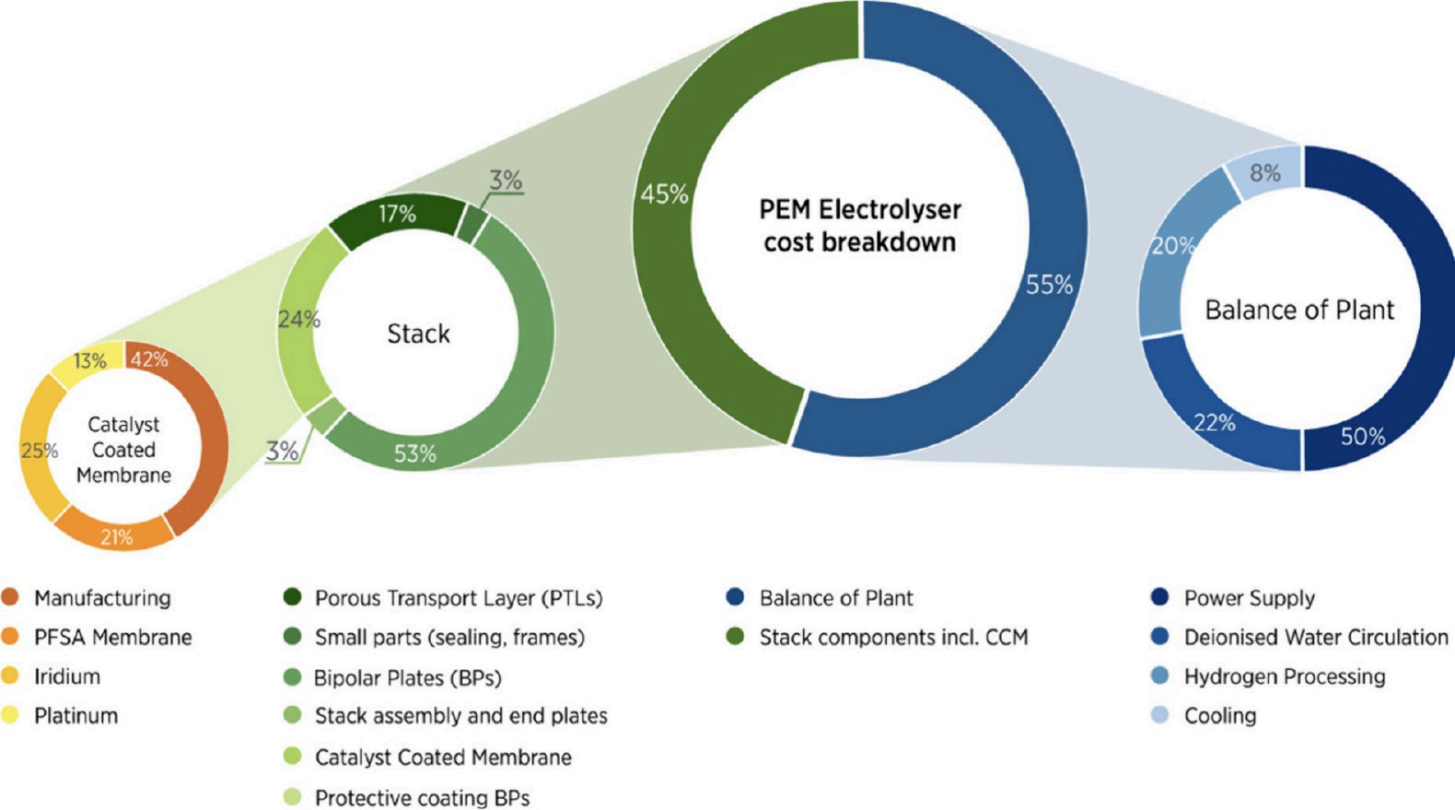
Cost distribution of a typical PEMWE stack. Reprinted with permission from ref (18). Copyright 2022, IRENA.

An electrolysis stack comprises several cells connected in series to electrically conductive BPs and end plates. When forming a PEMWE stack, homogeneous current distribution, water management, and suitable compression ratios must be provided in both the cells and stacks. In PEMWEs, water management is essential to achieve an equal lifetime in the performance of the stack and each cell. In stacks, water enters from the anode side, passes through all anode sides of each cell, and exits the anode sides of the end plate.^8^ Due to the electrochemical reactions occurring in the active surface area, the protons (H^+^) are transported to the cathode side through the membrane. Electrons are transmitted to the cathode side with the help of an external circuit, and hydrogen ions combined with these electrons form hydrogen gas at the cathode. The hydrogen gas formed is emitted from the cell by the cathode. Then between the cells the anode parts are connected to the cathode part (without any leakage). Water and oxygen are carried from all cells through these connections. Likewise, the cathodes of all cells are connected so that there is no leakage and the hydrogen outputs are given out through a single line. In the literature, there are many different studies on PEMWE stacks on reducing power fluctuations by integration with renewable energy sources of PEMWEs. For example, Joonas Koponen et al.^14^ analyzed the energy consumption and efficiency of a PEMWE stack by measuring the stack voltage, current, and hydrogen production at different hydrogen output pressures and power loads. To examine the effect of hydrogen output pressure on the specific energy consumption of a PEMWE stack, they tested hydrogen output pressures of 20, 30, and 40 bar at 25% load change. They concluded a significant increase in specific energy consumption with pressure change and less specific energy consumption under low-loading conditions. Selamet et al.^15^ developed PEMWE integrated with renewable energy sources. They first developed a single-cell electrolyzer with an active surface area of 50 cm^2^. They increased the electrolyzer performance from 74% to 87% with material and design improvements. Then they examined a five-cell stack that showed the performance effect of operating temperature and flow rate, and they obtained 80% efficiency at 1 A cm^–2^ and 50 °C. After that, they fabricated a 10-cell electrolyzer stack consuming about 1280 W of power. They noted that the performance of several stack cells did not change significantly. The hydrogen production flow rate of the electrolyzer stack is measured as 5 L min^–1^ and the current density is measured as 1.35 A cm^–2^. Mancera et al.^16^ studied the balance of subsystems for the PEMWE stack they integrated into renewable energy sources. The subsystems are discussed in five items: the collective power supply system, the water management system, the hydrogen generation subsystem, the cooling subsystem, and the control subsystem. The SCADA system provides monitoring of all subsystems, and data such as cell voltages, stack voltage, current, and power values can be monitored through this system. On the other hand, monitoring and emergency response of other subsystems, such as hydrogen drying and water management systems, can be performed via this interface. Krishnan et al.^17^ examined the cost effects of alkaline and PEM electrolyzer stacks from a 2030 perspective. Material cost is the most dominant contribution to direct cost for basic and advanced design. Stack components contributing the most to material cost are the membrane, BP, and electrodes for the basic design, as shown in Figure 3.

On the other hand, the critical components for advanced designs are membrane and BP. They stated that the most important driving force of the cost reduction envisaged in the advanced design is the switch to higher current density because if high current densities can be achieved, it will be possible to make the same production using less material. While the cost of PEM electrolyzers is 384–1071 €/kW for basic designs, this value can be as low as 63–234 €/kW for advanced designs. The highest impact on this cost belongs to membrane coatings and anode GDLs. In advanced designs, the most significant impact of cost reduction is to reduce the use of Pt and Ir by factors of 15 and 20, respectively.

### 2.1 Components of PEM Water Electrolysis Stacks

**Figure 4:**
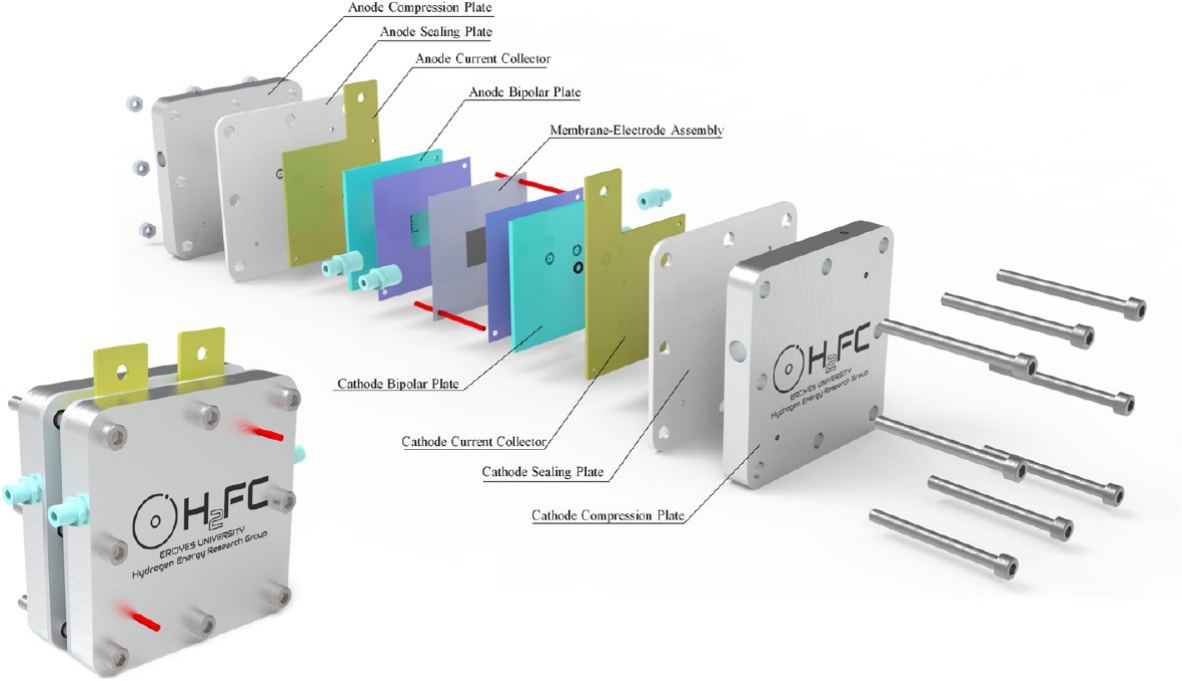
Overview of a typical single-cell PEMWE.

The primary PEMWE cells or stacks comprise several components such as the MEA, current collector, and separator plates. A typical overview of a single-cell PEMWE test setup can be seen in Figure 4.

The heart of the PEMWE cell comprises an MEA, which separates the cell into two half-cells (anode and cathode), separating the product gases, transporting protons, and supporting the anode and cathode catalyst layers. As seen in Figure 4, PEMWEs consist of compression plates, current collectors, flow channels, GDLs, and gaskets on the anode and cathode sides. Compression plates keep the cell’s equipment together and minimize contact resistance. Moreover, gaskets (not displayed) are used between the components to provide electrical isolation and prevent leakage. Current collectors provide the transmission of electricity supplied from the outside to the cell. When moving from a single-cell structure to a stacked structure with more than one cell, BPs are used instead of current collectors for the current collection process. BP has flow channels to ensure a uniform flow distribution over the GDLs and to allow the removal of the product gas. Flow channels allow the reactants entering the cell to reach the MEA. It can be preferred in geometries such as parallel, interdigitated, serpentine, mesh, and spiral to use the active surface area best. GDL is located between BP and MEA. It is used to distribute the fuel coming from the flow channels to the active surface area.

#### 2.1.1 Bipolar Plates

**Figure 5:**
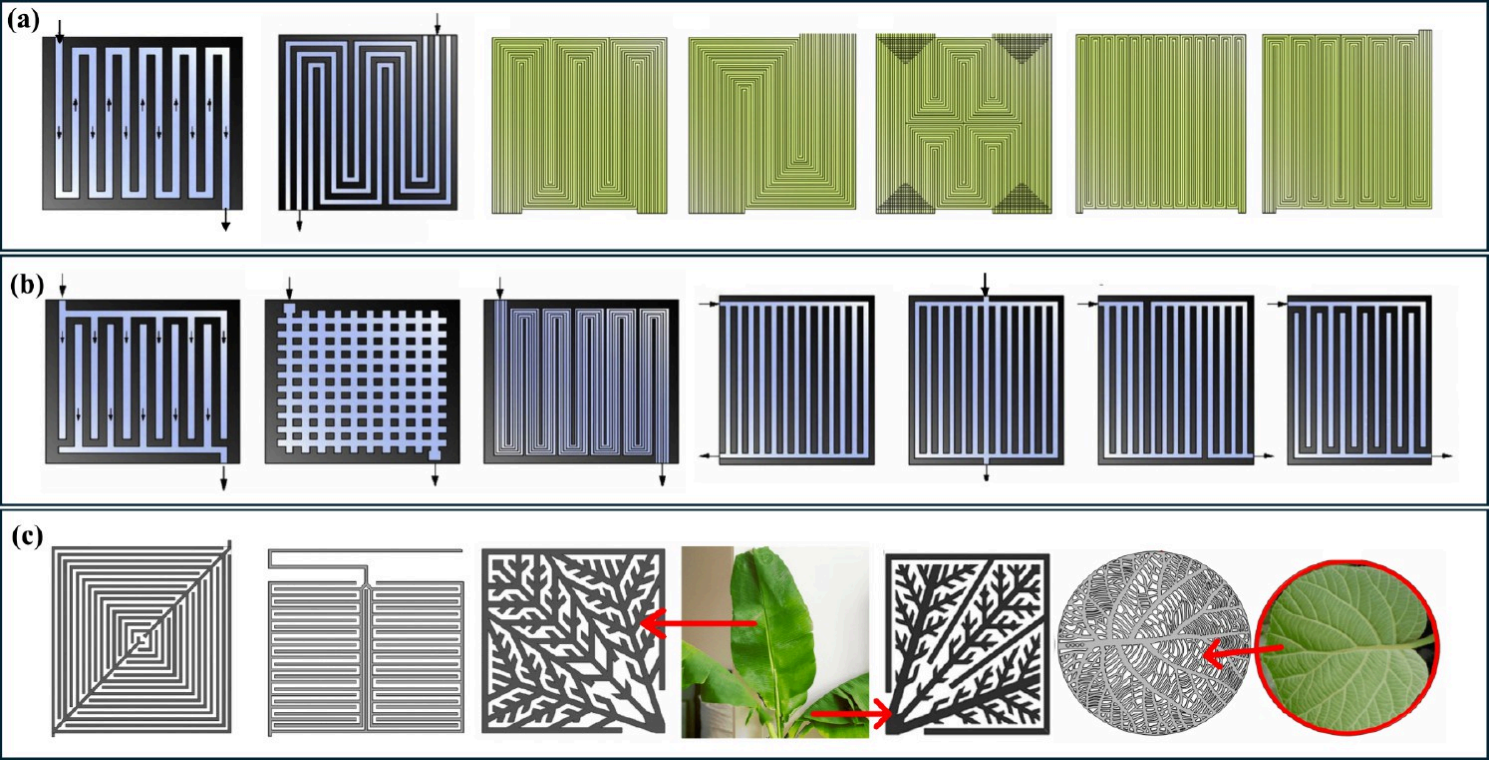
BPs geometry designs (by row): (a) serpentine, multiple serpentine; (b) parallel, pin, interdigitated design; (c) bio-inspired leaf and lung design. Reprinted with permission from refs (24 and 25). Copyright 2019, Elsevier.

BPs have essential tasks such as distributing the reactants inside the cell, removing the generated products at the end of the reaction through flow field distribution channels, removing electrons from the system, providing mechanical support to the cell, and there are different BPs geometries (see Figure 5).^19,20^ BPs are also expected to have high electrical conductivity, high corrosion resistance, high thermal conductivity, high strength, low gas permeability, low interfacial contact resistance, and low cost at PEMWE operation conditions.^21−23^ The cost of processing BP materials is an essential factor, because it will also affect the production costs of BPs during mass production.

**Figure 6:**
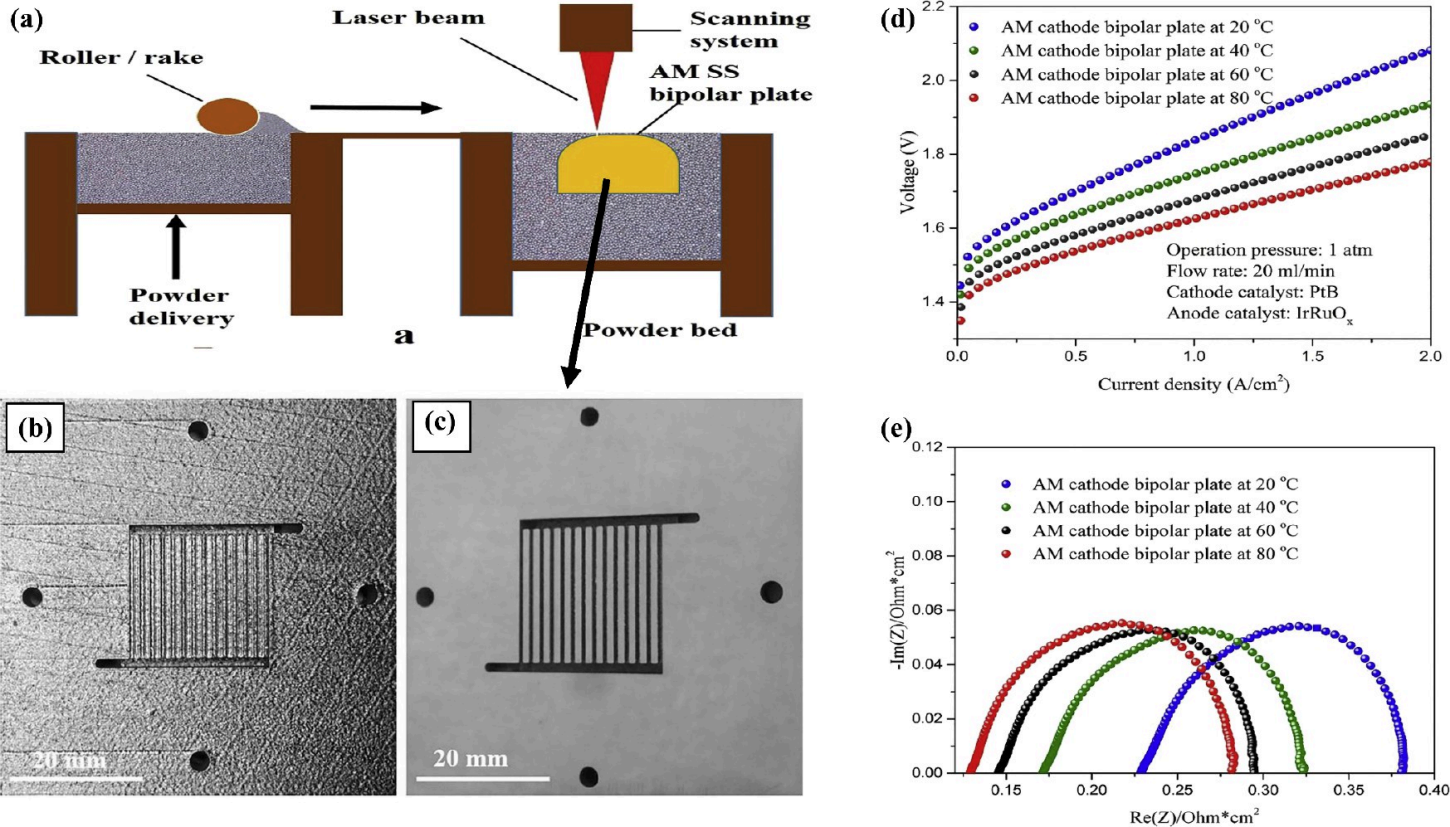
Schematic of high-efficiency BPs manufactured: (a) SLM technology, (b) before polishing, (c) after polishing, (d) polarization curves with AM SS cathode at different temperatures, and (e) EIS results with AM SS cathode at different temperatures. Reprinted with permission from ref (34). Copyright 2017, Elsevier.

Graphite BPs materials are the most suitable materials for the cathode side of PEMWEs with their high conductivity values and corrosion resistance.^26^ Also, carbon-based BPs are only preferred for the cathode side of PEMWE systems.^11^ However, when considering PEMWE systems, using carbon-based materials such as graphite as BPs at the anode side is more complicated because the anode side of PEMWEs has a more corrosive environment than the Polymer Electrolyte Membrane Fuel Cells (PEMFC). The applied voltage on the anode side of PEMWEs reaches up to 2 V, and the carbon-based or graphite-based materials typically start to oxidize at 1.8 V. This leads to a decrease in conductivity and lower performance in the PEMWE system.^27^ Metal-based materials such as Ti, stainless steel (SS), and Ni are used instead of graphite-based materials on the anode side of PEMWEs because of their high thermal and electrical conductivities, high corrosion resistance, and good chemical stability and durability.^28,29^ Among these metal materials, Ti-based structures are widely used as BP in PEMWE systems due to their excellent corrosion resistance, high thermal conductivity, low permeability, and low resistance properties.^30^ However, the surface of Ti materials is oxidized owing to the high operating voltage (1.8–2 V) and corrosive environment. Therefore, Ti-based BP materials are coated with different methods using noble metals such as Au and Pt.^31^ However, these coatings also increase the production costs of BPs. When PEMWE costs are analyzed, the overall cost of a cell consists of 24% of MEA and 48% of GDL and BPs.^6^ One of the biggest obstacles to spreading PEMWE systems is the high component and production costs.^32,33^ Therefore, it is necessary to investigate new materials and low-production methods for these systems to become increasingly common. In the literature, there are several studies that have reduced the costs of PEMWEs. For example, SS-based materials have come to the forefront due to their excellent corrosion resistance and high electrical conductivity. Yang et al.^34^ fabricated SS316L-based BPs for the cathode side of PEMWE using the additive manufacturing (AM) method which is also known as 3D printing. It is also noted that this study is the first example of cell testing in PEMWE systems (see Figure 6). As a result of the surface characterization analysis and in situ tests, it is revealed that metallic BPs can be produced using the AM method. The weight of the polished AM SS plate is 147.5 g, which is approximately half the weight of traditional graphite and copper plates. They reached a voltage of 1.779 V at a current density of 2.0 A cm^–2^ in in situ tests. They also stated that the decrease in the weight of BPs is minimal according to repeated SEM and SEM-EDX analysis after in situ tests. According to their results, it is emphasized that this study is a low-cost alternative and prototype to be produced in the PEMWE system.

Proch et al.^35^ investigated the availability of carbon-coated SS-based materials as BP in PEMWE. They are utilized in SS316L as the basis material, and the oxide layers on the surface of the SS materials have been cleaned by the plasma etching method. After the etching process, they coated the surface of SS materials by a physical vapor deposition (PVD) method using the carbon coating process. They analyzed the coated BPs in a single-cell system and preferred 60 °C temperature and ambient pressure for the tests. According to the tests’ results, the BPs are operated as stable for 720 and 1000 h on the anode and cathode sides, respectively. They also interpreted that the SS-based BPs would be an alternative to commercial Pt-coated Ti materials. Lettenmeier et al.^36^ prepared using SS-based material instead of Ti to reduce the production costs of PEMWEs. To increase the corrosion resistance of SS-based BPs, a titanium coating is made using the vacuum plasma sputtering method. Then, it was sanded with different grades of SiC for the Nb coating on Ti-based BPs. They used the magnetron sputtering PVD method for Nb coating of Ti-based BPs. According to the surface characterization results after the end of the coating processes, a 50-μm thickness for titanium coating and a 1-μm thickness for Nb coating have been obtained. Moreover, electrochemical measurements have shown that the corrosion resistance of BPs is increased and no corrosion is observed in the SS layer. When Nb-coated BP is compared with uncoated Ti-based BP, they noted that the Nb coating increases the interfacial contact resistance. It can be noted that the protective coatings do not significantly decrease the electronic conductivity of the cast SS. They stated that SS-based BPs could be used on the anode side of PEMWE systems using a commercial electrolyzer in 1000 h stability tests. Lettenmeier et al.^37^ coated the surfaces of SS316-based BPs using different metals for the PEMWE systems. The active surface area of the BPs is determined as 120 cm^2^, and coatings are made on both sides of the plates. SS-based BPs are first coated with 50–60 μm of Ti; then, Pt coating is made on the Ti layer (1.5 μm thickness). Vacuum plasma spraying and magnetron sputtering methods are used for the Ti and Pt coating process of BPs. After the coating processes, the BPs undergo 1000 h of long-term cell testing at a current density of 1 Acm^–2^. Although it is determined that the SS surface of the coating is wholly protected after the stability test, it is observed that there is degradation in the samples when used on the cathode side. Rojas et al.^38^ made coatings on different SS-based plates and compared them to reduce the costs of PEMWE systems. CrN/TiN, Ti/TiN, Ti, and TiN coatings are made on SS316L, SS904L, and SS321 using the cathodic arc evaporation method and a magnetron sputtering method. Before the coating process, the SS samples are sanded and cleaned in an ultrasonic bath. Mass losses and interface contact resistance (ICR) of the prepared BPs are compared to electrochemical measurements. As a result of the measurements, it is determined that SS321-based BP showed the best performance among SS-based materials. On the other hand, it is determined that Ti/TiN coatings have shown the best performance among coatings. In another study by Stiber et al.,^39^ instead of Ti used in BP and GDL, Nb/Ti coating on SS preserved its performance in the PEMWE cell, and a 13-fold increase in current density was achieved. The prepared coatings minimize gas formation between the interfaces and the anode, thus reducing the ohmic resistance and mass transfer losses. Lædre et al.^40^ have prepared BPs for use in PEMWEs using many different metal materials such as W, Ti, Ta, Nb, Mo, SS304L, and SS316L. They obtained the polarization curves of the prepared BPs and measured the ICR before and after the polarization experiments. As a result of the measurements, they stated that Ta-, Nb-, and Ti-based BP materials had obtained hydrogen at a lower current density than other BPs. On the other hand, it is determined that both Ta and Nb BPs have exceedingly small interfacial contact resistance compared to titanium-based BPs. Also, they noted that these materials did not undergo corrosion during mass measurements. According to weight measurements, it is noted that the mass increase of SS-based BP materials is around 20%, and these materials are more prone to corrosion. Finally, they noted this study as a guide for researchers on the preparation of BPs using varied materials. The authors held the study up as an example for other researchers to reduce the cost of PEMWEs. Table 1 shows the results of corrosion studies of BPs in the literature.

**Table 1:** *E*_corr_, *I*_corr_, and ICR Values of BPs in the Literature

Base Material	Surface Coatings	*E*_corr_ (mV)	*I*_corr_ (μA cm^–2^)	ICR (mΩ cm^2^)	ICR Pressure (N cm^2^)	Refs
Ti	TiN	–330	0.47	3	140	(41)
Ti	TiN	40	0.195	1.99	150	(42)
Ti6Al4V	TiN_*x*_O_*y*_	402.6	0.009	4.6	140	(43)
Ti6Al4V	TiN	80	0.22	6	140	(44)
Ti6Al4V	Ta_2_N	240	0.069	10.7	140	(45)
Ti6Al4V	TiSiN	–220	0.071	14.7	140	(46)
SS304	ZrN	–40	0.46	8.2	140	(47, 48)
SS304	Ti_3_SiC_2_	200	0.66	4.85	140	(48)
SS316L	TiN	–260	0.12	15.239	127	(49)
SS316L	Au/TiN	178	-	1.47	138	(50)
SS316L	Ti4O4-PPY	34	5.02	12.34	50	(51)
	PPY	–4	8.34	23.93	50	
SS316L	TiN	–213	0.099	1.544	150	(52)
	ZrN	31	0.209	18.86	150	

According to Table 1, it is seen that ICR tests are generally performed at a pressure value of approximately 140 N cm^2^. There is only one study that found that 50 N cm^2^ is the preferred pressure for the ICR. The highest ICR value is measured in this study. SS316L is chosen as the base material, while PPY was selected as the surface coating. The ICR value was measured at 23.93 mΩ cm^2^ under a compression value of 50 N cm^2^. In the study where the lowest ICR result (1.47 mΩ cm^2^) is observed, SS316L is again used as the base material. However, Au/TiN is preferred as the coating material. These two studies demonstrate that the coating material is the crucial parameter for the ICR, rather than the base material. Developing new material technologies for PEMWEs, using durable, efficient, and inexpensive components can improve electrolysis performance.^53^ At the same time, thanks to the developing material technologies, there have been performance improvements by using lighter and less expensive materials, and their use has become possible in different working areas.^54^ It can be seen that titanium is frequently preferred in BPs. Although it continues to be used for the high corrosion resistance and high potential strength provided by Ti layers, it is an expensive metal, which is an obstacle to the commercialization of this technology.^55^ In recent years, studies have been conducted on the availability of various materials in addition to Ti, with a quality that can replace Ti. Material technology of BPs can be one potential research area that can increase the performance of PEMWE systems. In addition, reducing the production costs of BPs is seen as a significant gain. So, new researchers need to work on advanced material technology for BP.

#### 2.1.2 GDLs

**Figure 7:**
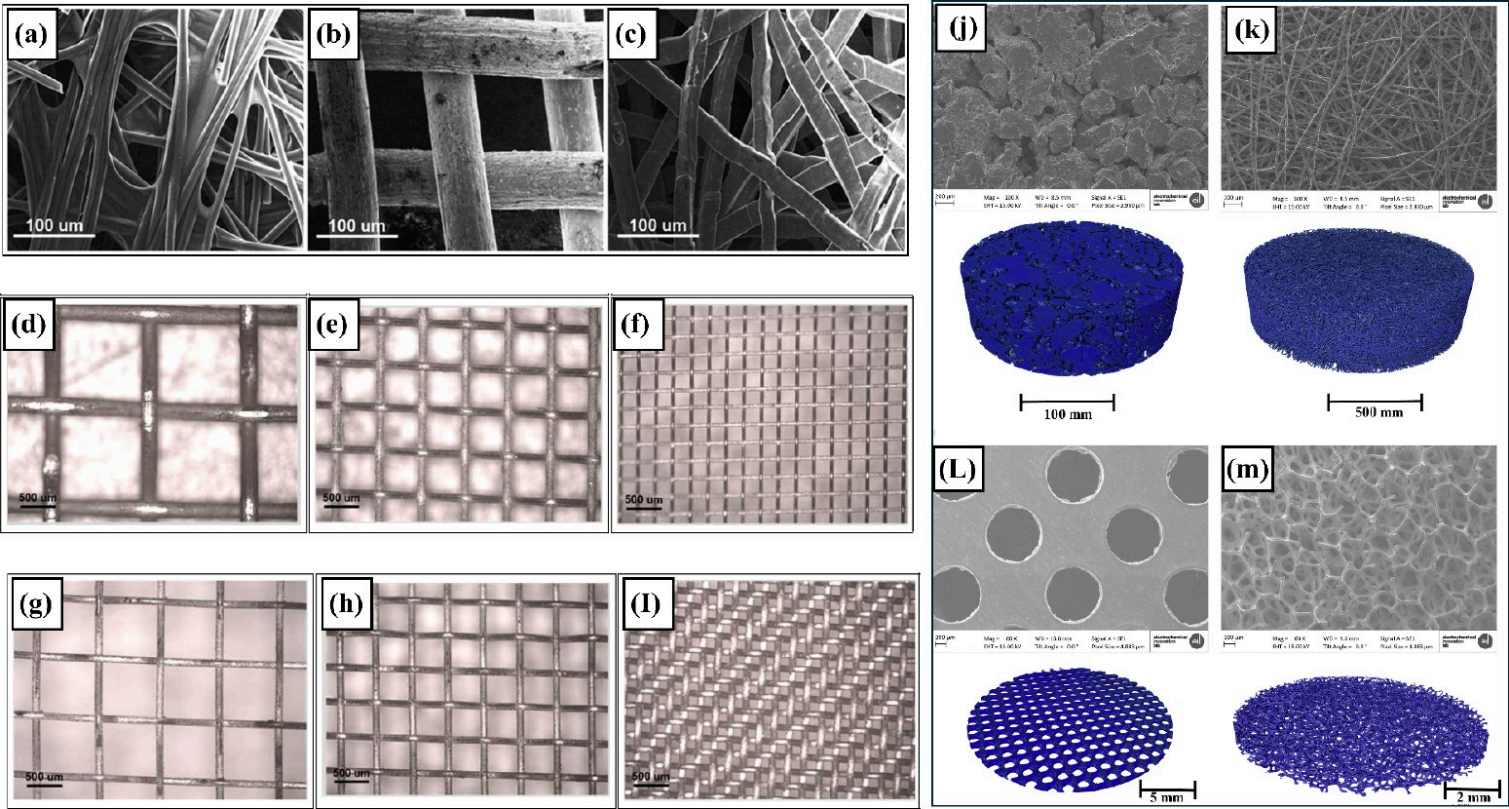
SEM images of GDLs: (a) Toray-090 carbon paper, (b) titanium mesh, (c) titanium felt, and illustration of titanium GDL mesh for various thickness: (d) 534 μm, (e) 278 μm, and (f) 170 μm along with various porosity values; (g) 0.77, (h) 0.62, (i) 0.27 reproduced using ref (64). SEM images (gray) and X-ray Tomograms (blue) of various GDL materials: (j) sintered powder, (k) fibrous materials, (l) perforated plates, and (m) metal foams. Reprinted with permission from ref (65). Copyright 2022, Elsevier.

GDLs can be used with a porous catalyst layer and diffusion media to facilitate the transport and homogeneous distribution of the reactants. GDLs are widely used in other electrochemical energy conversion devices such as electrolyzers, electrochemical compressors, and fuel cells, which are optimized for high current density and low mass losses. In PEMWE systems, the efficiency is directly proportional to the amount of water that reaches the catalyst-coated membrane.^56,57^ The porosity ratio value in the anode GDLs must be carefully determined to regulate mass transfer. GDL should have a high porosity value that does not slow the water flow rate. However, at values more significant than the required porosity value, it is caused that the water not to be appropriately distributed over the active surface area.^58−60^ Since the anode side of PEMWEs has a highly oxidizing and corrosive environment, corrosion causes an increase in contact, ohmic, and activation losses, so cell efficiency is decreased.^61,62^ Therefore, materials and coatings with high corrosion resistance are used in this component of the PEMWEs.^13^ Generally, Pt-coated Ti mesh structures are preferred as GDL on the PEMWE anode side.^63^ In particular, developing alternative electrodes to Ti materials, which are expensive but have high corrosion resistance in acidic environments, is critical for better recognizing and commercializing PEMWEs. For example, Steen et al.^64^ used titanium GDL with different thicknesses and porosity values in the PEMWE cell. They used carbon paper (Toray-090) as the GDL on the cathode side of PEMWE. On the anode side, Ti mesh structures and Ti felt structures are used to compare the effects of thickness and porosity (see Figure 7). Both galvanostatic and potentiometric measurements have been conducted better to understand the connection between ohmic and mass transfer losses.

As a result of the tests, it is determined that the thickness and porosity values play a significant role in cell performance. In addition, it is observed that decreasing the thickness reduces both the ohmic resistance and the mass transfer losses in the samples with the same porosity value. Thus, it has been stated that the cell performance increased.

Cruz et al.^66^ investigated the morphological effects of modified Ti matrix GDL samples. They examined the microstructural effects of samples on mass transport using numerical simulation and statistical electrochemical techniques. As a result of the analysis, it is determined that the treatment increased the GDL performance for liquid distribution and provided faster electrical conduction than a porous Ti matrix. At the same time, according to the results of SEM and EDX analysis, they interpreted that the fluid mass transfer is enhanced and determined that there is no homogeneous distribution of small particles and high porosity. Borisov et al.^67^ studied improving new GDLs suitable for PEMWEs. They used GDL with stoichiometry Magneli phase titanium oxide instead of carbon-based GDL. They then integrated their developed GDL into an MEA containing an active Pt catalyst and a proton-conducting PEM. The results showed that the electrode has an insufficient porosity for optimizing the GDL preparation method by using the Pt catalyst. Kıstı et al.^68^ used cost-effective commercial SS316 mesh structures as GDL for PEMWE. They coated SS316 mesh with Ni–Mn_*x*_O_*y*_ to increase the catalytic activity of commercial mesh structures. They conducted experiments to determine the electrochemical properties of the obtained GDL. As a result of the experiments, they determined that the coating improved the electrochemical properties of GDL. Finally, it is mentioned that commercial mesh structures coated with Ni–Mn_*x*_O_*y*_ can be an inexpensive alternative to PEMWE. In another study, Kıstı et al.^60^ examined Pt-coated SS316L structures as an inexpensive alternative for use in PEMWEs. They first performed pressure tests on commercially available structures with different porosity values and four different mesh structures. Then they determined the best sample with the best pressure drop. To increase the corrosion resistance of the obtained sample and improve its electrocatalytic properties, they coated samples with Pt at different molar ratios. According to the obtained results, it is stated that the corrosion resistance of the sample increases by the Pt content in the plating bath. Table 2 summarizes the GDLs used for the anode side of the PEMWE systems in the literature.

**Table 2:** Comparison of PEMWE GDLs Studies in Literature

Base Material	Surface Modification	Anode Loading	Cathode Loading	Current Density	Refs
Ti	Ir coating	2.2 mg·cm^–2^ IrO_2_	0.8 mg·cm^–2^ Pt	2 A.cm^–2^@1.857 V	(69)
				1 A·cm^–2^@1.674 V	
TiO_2_	IrO_2_ coating	3.0 mg·cm^–2^ IrRuO_*x*_	3.0 mg·cm^–2^ Pt Black	2.824 A·cm^–2^@2.0 V	(70)
Ti	IrO_2_ coating	1.5 mg·cm^–2^ IrO_2_	0.5 mg·cm^–2^ PtC (60%)	2.400 A·cm^–2^@2.1 V	(71)
Ti	Ir_0.7_Ru_0.3_O_2_ coating	1.0 mg·cm^–2^ Ir black and 2.0 mg·cm^–2^ Pt	0.4 mg·cm^–2^ Pt	2 A·cm^–2^@1.848 V	(72)
	(Ir_0.7_Ru_0.3_)Ta_0.1_O_2_ coating			2 A·cm^–2^@1.851 V	
	(Ir_0.7_Ru_0.3_)Ta_0.3_O_2_ coating			2 A·cm^–2^@1.836 V	
	(Ir_0.7_Ru_0.3_)Ta_0.5_O_2_ coating			2 A·cm^–2^@1.899 V	
	(Ir_0.7_Ru_0.3_)Ta_0.7_O_2_ coating			2 A·m^–2^@2.026 V	
	Pt coating			2 A·m^–2^@1.87 V	
Ti	-	2.2 mg·m^–2^ IrO_2_	0.8 mg·m^–2^ Pt	0.8 A·m^–2^@2 V	(73)
	Ir coating			2.8 A·m^–2^@2 V	
	Pt coating			2.85 A·m^–2^@2 V	
	Au coating			1.4 A·m^–2^@2 V	
Ti	-	3.0 mg.cm^–2^ IrRuO_*x*_	3.0 mg.cm^–2^ Pt Black	2 A.cm^–2^ @ 1.685 V	(74)
	Au coating			2 A.cm^–2^ @ 1.632 V	
Ti	Nb/Ti coating	2.5 mg·m^–2^ IrO_2_	0.95 mg·m^–2^ Pt	2 A·m^–2^@1.98 V	(75)
SS				2 A·m^–2^@1.97 V	
SS	-	3.0 mg·m^–2^ IrRuO_*x*_	3.0 mg·m^–2^ Pt Black	1 A·m^–2^@2.8 V	(62)

In Table 2, GDL studies in the literature can be seen. When the table is examined, it is seen that carbon-based materials are not preferred on the anode side of the PEMWE cells. Instead of carbon-based structures, metal-based materials are used on the anode side that can withstand a highly corrosive environment. It is seen that Ti materials are generally preferred as the base materials. The highest current density was obtained using Pt-coated Ti GDL as 2.8 A·cm^–2^@2 V. It was observed that Pt-coated Ti GDL was followed by Ir-coated Ti and IrO_2_-coated TiO_2_ GDL with current density values of 2.8 A·cm^–2^@2 V and 2.824 A·cm^–2^@2 V, respectively. However, the search for alternative materials also continues for GDL. Despite its features, such as being easier to process, accessible, and cheaper than Ti structures, SS products that can withstand the corrosive environment on the anode side come to the fore. Stibe et al.^75^ found that almost the same power values were obtained by changing only the base material. This shows that using SS material, a cheaper option in PEMWE systems, will reduce system costs. For this reason, SS materials can be preferred as a cheap alternative to Ti as the primary GDL material.

#### 2.1.3 Membrane Electrode Assembly

A high-performance MEA designed for PEMWE should have features like exhibiting robust adhesion among the catalyst and membrane, three-phase boundaries (TPB) where the electrolyte and catalysts come into contact for the reaction to occur, low resistivity between the CL and the membrane, and easy release of gas bubbles. Moreover, other factors such as chemical stability, mechanical durability, and cost-effectiveness are important considerations in the design and development of high-performance MEAs for PEMWE applications. Currently, the preparation of MEA for PEMWE is very similar to the preparation procedure for PEMFCs. The majority of MEAs are prepared using the spraying method (known as catalyst-coated membrane (CCM) and catalyst-coated GDL (CCG) method), by which catalysts are sprayed directly onto a membrane or a backing/current collector substrate.^76,77^ Briefly, optimizing the MEA design for PEMWEs involves a combination of material selection, fabrication techniques, and performance testing to meet the specific requirements of electrolysis applications, considering factors such as durability and low cost-effectiveness. The central component of PEMWE is the MEA, which consists of a PEM and CLs. The membrane is the most crucial component of a PEM cell, exerting a substantial influence on the purity of the generated gases and the system’s durability. The membrane acts as a barrier to prevent the mixing of hydrogen and oxygen gases produced at the anode and cathode, thus ensuring the purity of the gases. This is essential for high-purity hydrogen applications such as fuel cells, electrolyzers, or industrial processes. A high-quality membrane ensures the longevity of the PEM cell by withstanding harsh operating conditions such as high temperature, high humidity, and an acidic environment. It also resists chemical degradation and mechanical stresses, which are critical for system durability. Sulfonic acid groups are commonly used as functional groups in PEMs. The combination of these polar groups with the hydrophobic aromatic backbone gives the membrane a phase-separated structure.^78^ The most commonly used membranes in PEM applications are perfluorosulfonic acid (PFSA) polymer membranes such as commercial Nafion membranes (N115, N117), Aquivion (Solvay-Solexis), Fumion (FuMA-Tech), Aciplex (Asahi Chemical), and Flemion (Asahi Glass).^79,80^ Among these membranes, Nafion and Aquivion membranes have the important properties of high proton conductivity (0.09–0.11 S/cm at 80 °C and 100% RH) and high mechanical strength (∼20 MPa). These membranes have properties suitable for use as solid electrolytes, providing excellent thermal and chemical stability, high mechanical resistance, and high proton conductivity.^81^ On the other hand, it has disadvantages, such as high cost, presence of fluoride in the polymer structure (which can migrate and cause corrosion in the metallic elements of the electrolyzer), mechanical behavior at high temperatures and thickness (which increases ohmic resistance and decreases performance at high current densities).^82,83^ Moreover, hydrocarbon membranes such as polybenzimidazoles, poly(ether sulfones) (PES), sulfonated polyphenyl quinoxaline, and poly(ether ether ketones) (PEEK) have also been improved to reduce the cost of membrane materials.^84^ Membranes are expected to have durable and flexible properties. At the same time, membranes should have many features, such as high efficiency, not undergoing dimensional changes in temperature, high stability, and good proton conductivity. Nafion membranes with different thicknesses (Nafion 211, 212, 115, 117, etc.) are widely used in PEMWE applications.^85,86^ Perfluorosulfonic acid polymer membranes such as Nafion are highly prone to operate at high current densities (2 A cm^–2^), and they stand out among other membrane types with their high strength, good proton conductivity, and mechanical stability.^10^ These materials have a proton conduction mechanism.^87^ Building on this established technology, a new composite membrane has been developed by combining glass fiber-reinforced PFSA with sulfonated syndiotactic polystyrene (ssPS) as a potential alternative to conventional Nafion membranes.^88^ These composite membranes are integrated into MEAs by using substrates coated with catalysts that are deposited through electrochemical methods. Both individual components and complete MEAs were evaluated through ex situ characterization and in situ testing in PEMWE conditions. Initial performance tests showed that the novel MEAs achieved a voltage of 2.0 V at a current density of 0.5 A cm^–2^. However, compared to Nafion membranes in PEMWE, other membranes such as Fumapem, Flemion, and Aciplex are not preferred because they are not resistant to high current densities and are weaker regarding mechanical stability.^89^ On the other hand, to enhance PEM performance, a systematic purification and functionalization process is essential. The procedure involves two main steps: surface oxidation and protonation. The oxidation phase employs either concentrated HNO_3_ (approximately 35 wt %) or H_2_O_2_ solution (around 5 wt %) to remove surface contaminants.^90,91^ During this step, PEMs are immersed in the oxidizing solution and heated to 80 °C for 1 h. Following oxidation, the PEMs undergo a thorough cleansing process using deionized water at the same temperature and duration to remove any residual oxidizing agents.^92^ The second key step involves treating the PEMs with a 1 M H_2_SO_4_ solution, which serves to increase their proton conductivity. This acid treatment is also conducted at 80 °C for 1 h. A final rinse with deionized water removes any loosely bound protons from the PEM surface, resulting in a properly functionalized PEM ready for application.

Considering the PEMWE components in recent years, although it is seen that the studies are primarily focused on BP and catalysts, there are also studies on other components.^93,94^ Different polymeric structures continue to be developed as alternatives to Nafion membranes. However, experimental studies continue for these new membranes to replace Nafion completely. Therefore, searching for improved membrane materials is open for scientific and industrial scale studies. The membrane types used in previous studies in the literature and the coating materials used in the anode and cathode are given in detail in Table 3

**Table 3:** Diverse Types of Membranes and Catalyst Coating Materials with Loading

Membrane	Active area (cm^2^)	Preparation method	Anode Catalyst Coating Material	Cathode Catalyst Coating Material	Thickness (μm)	Anode Catalyst Loading (mg·cm^–2^)	Cathode Catalyst Loading (mg·cm^–2^)	GDL	Cell Voltage (V)	Refs
Nafion 117	36	CCM	Ir	Pt/C	179	2	1	Ti	-	(95)
Nafion 117	25	CCM	IrO_2_	Pt/HSC	-	0.55 ± 0.03	-	Carpon paper	-	(96)
Nafion 115	-	CCM	Ir	Pt	-	2.5	0.95	Ti-mesh	-	(39)
Nafion 117	-	Decal transfer	IrO_2_	Pt/C	180	2.0	0.4	Carpon cloth	1.35 and 1.9	(97)
Nafion 115	4	CCM	Ir	Pt/C	-	0.14	0.4	-	2.0	(98)
Nafion 117	25	CCM	IrO_2_	-	175	1.31	-	-	-	(99)
Nafion 117	25	CCM	Ir Black	Pt/C	-	2	0.4–2	Ti	1.77 and 2.10	(100)
Nafion 115	25	CCM	IrO_2_	Pt	125	-	-	Ti	-	(101)
Nafion 115	4	CCG	IrO_2_	Pt/C	115 ± 12	1.5	0.5	Ti-felt	1.8	(102)
Nafion 212	9	CCM	-	Pt/C	-	-	0.5	Ti	1.83	(103)
Nafion 117	4	CCG	P/M/RGO	Pt/C	-		0.5	-		(104)
Nafion 212	25	CCM	Ir	Pt	-	2	1	Ti		(105)
Nafion 117	-	CCM	-	Pt/C	175	-	-	-	-	(106)
Nafion 115	-	CCM	IrO_2_	Pt/C	-	7.5	-	Ti-sheet	1.7	(107)
Nafion 117/115	5	CCM	IrO_2_	Pt	-	3	0.4	Ti-mesh	-	(108)
Nafion 115	-	-	IrO_3_	-	-	-	-	-	-	(109)
Aquivion	5	CCG	IrO_2_	Pt/C	120	2	0.3	Ti and Carbon cloth	1.73	(110)
Aquivion E87–12S	6.25	CCM	IrO_2_	Pt	120	2	2	Carbon paper	1.76	(111)
PFSA/ssPS	25	CCM	IrO_*x*_/ATO Ti-PTL IrO_*x*_/ATO	Pt/carbon nanofiber	63.5 ± 4.5 and 107.5 ± 6.8	1	0.4	Ti-PTL and carbon paper	2.84 and 2.60	(88)

**Figure 8:**
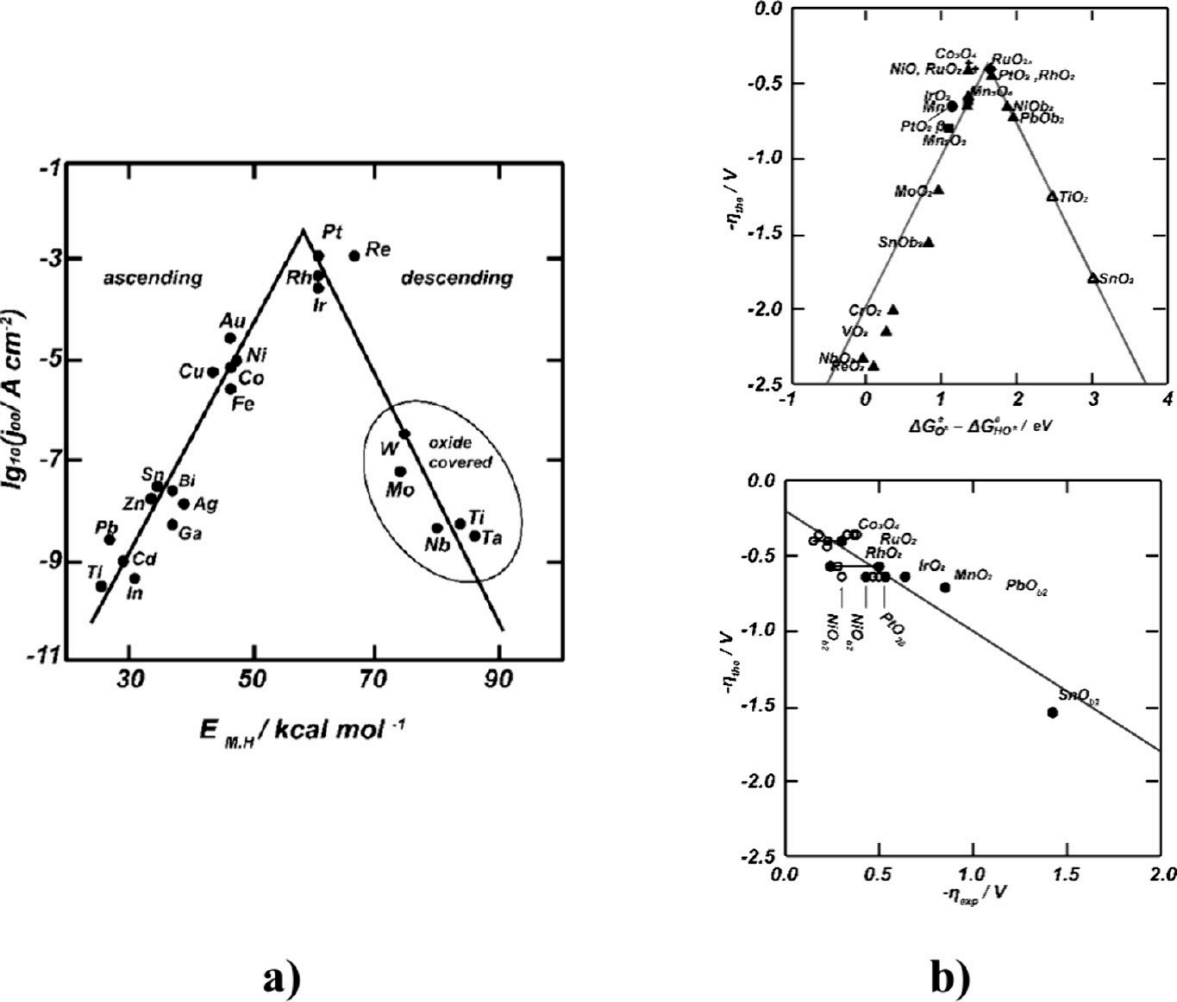
Volcano plots for (a) HER and (b) OER vs (Δ*G*_O*_ – Δ*G*_HO*_). Reprinted with permission from ref (118). Copyright 2011, Beilstein.

As seen in Table 3, Nafion 115 and 117 are generally preferred in PEMWE studies. It is necessary that the corrosive effect of PEMWE does not damage the membrane in the reactions taking place at the anode and cathode and that a long-life proton-permeable material can be obtained. It can be seen that the thickness of the membrane is shaped around the operating conditions of the system. Preferred membrane thicknesses in common use vary between 25 to 200 μm. When choosing the membrane, the current density of the system, the hydrogen production rate, and the system life should be evaluated for the most appropriate thickness, considering the material type and the system’s performance to be applied. Although thin membranes can provide a higher proton conductivity and better performance, they are susceptible to mechanical damage and deterioration. Thicker membranes have higher mechanical stability and durability, increasing the internal resistance and reducing the overall system efficiency.^112^ For optimum membrane selection, membranes of appropriate thickness for intracellular performance should be chosen by considering the balance among proton conductivity, mechanical stability, and system efficiency. In the electrochemical reactions in PEMWE, catalysts are the main factor influencing the reaction rate and efficiency. The absence of electrolyzer catalysts results in high overpotentials, low electrode reactions, and poor kinetic activity. Catalysts positively affect the hydrogen production process, helping to reduce the input voltage and offering the advantage of precise intracellular control. The energy efficiency of the cell is directly related to the overpotentials at the anode and cathode sides.^113^ As the cell’s driving force, OER and HER provide very high-purity oxygen and hydrogen production.^114^ Electrocatalyst varieties are limited around Pt group elements (Pt, Ir, and Ru) due to a low pH environment and high oxygen concentration. In PEMWE, Ir-IrO_2_, Ru-RuO_2_, and Pt, Pt/C catalysts are widely used on the anode and the cathode side, respectively. These expensive catalysts are preferred because of their high corrosion resistance and catalytic activity.^115^ Using non-noble metals as electrocatalysts in an acidic environment is not preferred due to oxidizing rapidly and dissolving in solid electrolytes.^116^ Although high catalyst loading is commonly used in PEMWE applications to eliminate the high costs, which is an essential problem in applications, it is offered as an option to reduce the amount of loading or replace the expensive noble metals used in the catalyst layers.^89,117^ Recently, decreasing Pt loadings and including non-noble conductive materials (such as C) in catalyst studies have contributed exceptionally to reducing HER costs. While the Pt loading amount is between 0.4 and 0.6 mg cm^–2^ on the cathode side,^11^ Ir loading on the anode side is around 0.2 to 3.0 mg cm^–2^.^115^ In PEMWE studies, generally, noble-metal oxides are used to challenge the oxidative environment of the OER reactions. Volcano plots, a functional expression of stability and activity, are shown in Figure 8 best to describe the HER and OER catalyst kinetics for PEMWE.

**Figure 9:**
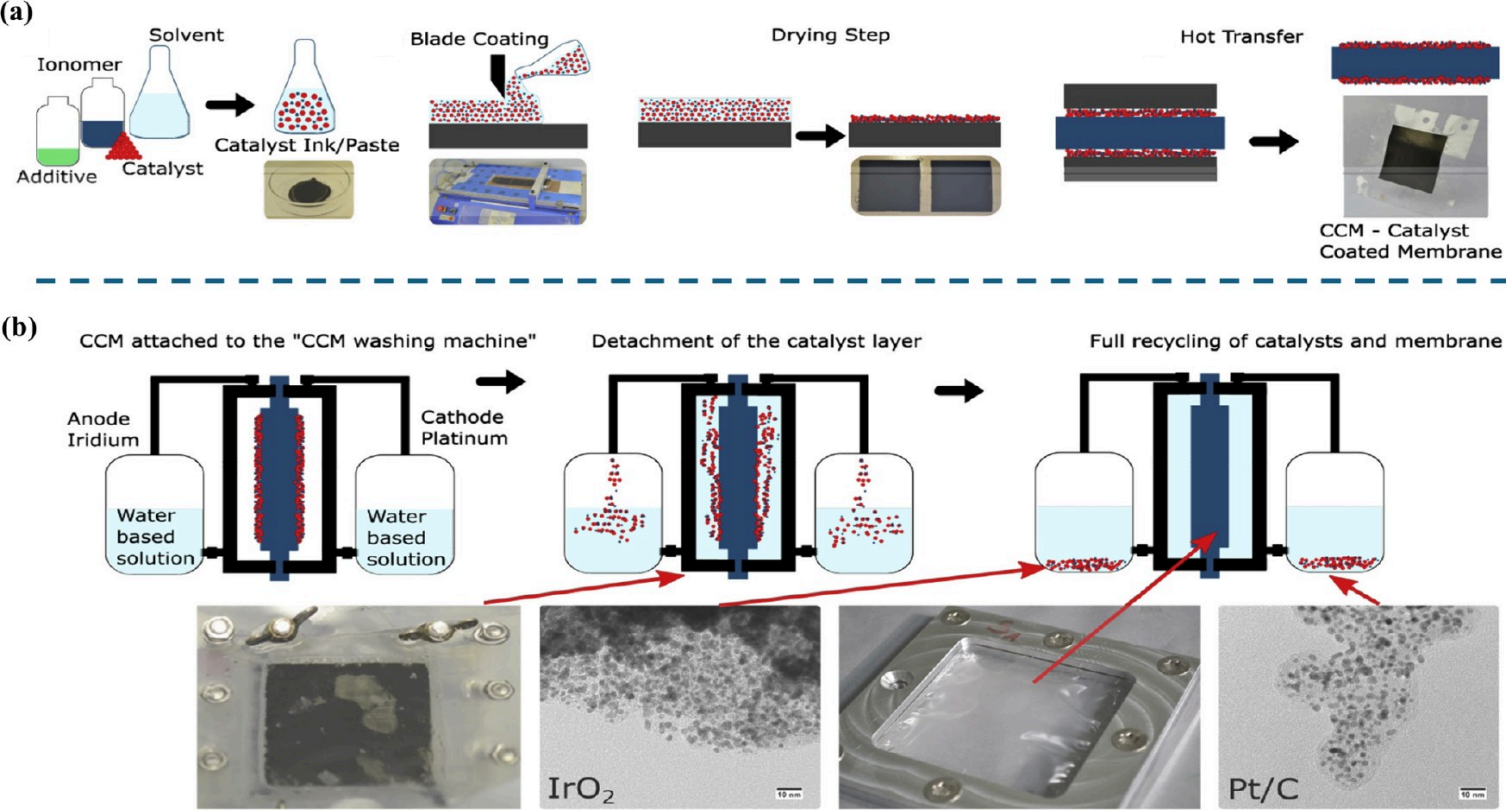
(a) Steps for CCM fabrication and (b) CCM recycling. Reprinted with permission from ref (128). Copyright 2019, Elsevier.

HER are reversible reactions that take place in more than one step. HER catalysts effectively reduce protons to hydrogen at the negative electrode. They increase the reaction rate by reducing the activation energy.^119^ The catalytic activity of Pt, Pd, and Ni, which is frequently preferred for HER in acidic environments, with other metals is as follows: Ni < Ru < Os < Re < Ir < Rh < Pt < Pd.^115^ The Sabatier Principle^120^ explains that Pt has the best catalyst activity. This principle is a description of the differences in catalytic activity between the catalysts. The accumulation of precious metals at the top and the spread of other metallic catalysts to the lower arms can be better understood by examining the Volcano plots (Figure 8a). It is known that on the right side of the Volcano plot given in Figure 8a, elements are more oxidized than others during HER. These oxides also can reduce the reaction rate, causing undesirable conditions on the cathode side.^118^ Pt is located at the top of the volcano curve due to its high catalytic activity.^121,122^ Due to the high anodic potential of PEMWE, carbon or carbon-based materials can often be used at the cathode.^115^ It is known that 40% by weight Pt/C is present in commercial HER catalysts. Although reducing the Pt catalyst content with carbon provides a cost advantage, alternative catalysts equivalent to Pt catalysts are still under investigation. At the same time, Pt is considered the most useful noble metal in preventing complex HER reactions.^11^ As can be seen in the Volcano plot for OERs (Figure 8b), the most active oxides are Au ≪ Pt < Ir < Ru ≪ Os monometallic catalysts, but the least stable are Au≫ Pt > Ir > Ru ≫ Os materials, respectively.^123,124^ According to the Sabatier Principle,^125^ a volcano plot of η_OER_ vs (Δ*G*_O*_ – Δ*G*_HO*_) might be produced in this scenario, with the ideal value of Δ*G*_O*_ – Δ*G*_HO*_ being about 1.6 eV. Therefore, Ir and Ru appear to be the most helpful catalyst materials. The increased frequency of Ir 5d-band holes and shortened Ir–O ligand bonds, which lead to the creation of electrophilic O^(II−δ)–^ surface species and favor the nucleophilic attack of water, are responsible for the effective OER performance of Ir-based catalysts.^126,127^ Various methods of catalyst synthesis and measurement ranges of catalyst materials have been extensively studied to improve the catalyst performance. For example, Carmo et al.^128^ studied the recycling of precious metals used in catalysts (membrane/catalyst separation). They emphasized that when the investment cost in PEMWEs is considered, the costs of catalysts can be reduced by reusing (recycling) the catalyst materials, as shown in Figure 9.

The amount of coating on both the anode and cathode sides and the catalysts used in previous studies in the literature are listed in detail in Table 4.

**Table 4:** Loading Amounts of Anode and Cathode Catalysts with Different Catalyst Synthesis Methods

Type of Synthesis	Anode Coating Material	Cathode Coating Material	Anode Loading (mg cm^–2^)	Cathode Loading (mg cm^–2^)	Types of Analysis	Range of Measurement	Ref
Electrodeposition Method	Ir@WO_*x*_NR	Pt	9–144 μg cm^–2^	0.2	EIS	10 kHz/0.1 Hz	(98)
					LSV	-	
					CV	0.2/1.2 V_SCE_	
Solvothermal Method/CVD Method	MTMs-OS NW	-	-	-	EIS	100 kHz/0.1 Hz	(129)
					LSV	0/1 V_(vsAg/AgCl)_	
					CV	–0.2/0.6 V_(vsAg/AgCl)_	
Adam’s Fusion Method/Magnetization Method	IrO_2_/Fe_3_O_4_	Pt	3	0.4	EIS	-	(108)
					LSV	0/1.7 V_(vs Ag/AgCl)_	
					CV	-	
Thermal Decomposition Method	IrO_2_–Fe_2_O_3_	-	0.125	-	EIS	100 kHz/0.1 Hz	(130)
					LSV	–0.2/0.2 V_(vsRHE)_	
					CV	0/1.4 V	
Adam’s Fusion Method	WO_3_Ir_*x*_Ru_1–*x*_O_*x*_	Pt/C	1–6	>1	EIS	-	(131)
					LSV	-	
					CV	1.2/1.7	

As seen in Table 4, the electrodeposition method has the lowest anode and cathode loading amount compared to the other catalyst synthesis methods. In this study by Jiang et al.^98^ the fact that Ir is an expensive material with limited reserves has inspired a search for alternative catalysts that can be used in OER. With the anode catalyst studies synthesized in composite form, it has been stated that composites are cheaper and more stable. Thus, solvothermal, chemical vapor deposition (CVD), Adam’s Fusion, magnetization, and thermal decomposition methods continue to develop composite catalysts. When the analysis types are examined, it is seen that the determined measurement intervals vary, consistent with the preferred reference electrode and material. In some studies,^108,129^ it is seen that the catalyst can be synthesized by combining the two synthesis methods. In Khiarak et al. when the analysis types are examined, it is seen that the determined measurement intervals vary in line with the preferred reference electrode and material. In some studies, it is seen that the catalyst can be synthesized by combining the two synthesis methods. In Khiarak et al.,^129^ the CVD method is used together with the solvothermal method. Different 3D catalysts were synthesized by preparing graphene nanosheets. This OER study in an alkaline environment has been added as a methodical example. When the developments in material technologies are examined, it is frequently seen that studies are gathered around expensive platinum group metals (PGM). Thus, the cost barrier in the PEMWE technology is also encountered by catalysts. In the highly corrosive acidic environment of PEMWE, IrO_2_ and Pt are used as catalysts on the anode and cathode side, respectively. At the same time, IrO_2_–RuO_2_ catalysts can be used instead of IrO_2_ for the OER on the anode side of PEMWEs. There are also studies in which these two catalysts are used together y coating IrO_2_ as a protective layer on RuO_2_.^132,133^ In the study of Cha et al.,^134^ IrO_*x*_/Ti_4_O_7_ catalysts were synthesized at different rates in order to minimize the amount of Ir loading in MEA. It has been experimentally proven that the IrO_*x*_/Ti_4_O_7_(7:3) catalyst they prepared is the catalyst with the best Ir/support ratio in MEA. Thus, the most optimal result of catalytic stability, catalytic activity, and corrosion resistance has been obtained. Based on the study, it is understood that reducing the Ir loading amount helps to establish a more efficient and cost-effective PEMWE system. It is seen that more than one material mixture can be used as a catalyst to increase the electron conductivity and mass transfer. In another study of composite catalysts, Uzgören et al.^135^ have the first to obtain WO_3_ powder for use in OER by recycling waste wire drawing dies. They demonstrated the electrochemical recycling of WC–Co wire drawing dies by synthesizing an IrO_2_–WO_3_ mixed metal oxide catalyst as an excellent alternative to expensive IrO_2_. They reduced the cost of the OER catalyst by approximately 25% and improved its catalytic activity. It has been suggested that using recycled materials in energy conversion devices could be an excellent solution to the cost of catalysts, which is one of the barriers to commercializing these technologies.

## 3 Stacks of PEMWE

In the PEMWE stack, several single cells are connected in series to obtain higher hydrogen amounts and greater power values. Obtaining a higher pressure to lower the compression energy is another crucial factor for stack studies. Electrochemical compression in PEMWE stacks provides pressurized hydrogen gas, eliminating the need to process high-pressure oxygen. Hydrogen must be produced or compressed at high pressure, like 700 bar, to store hydrogen in pressurized cylinders.^136^ While PEMWE systems seem like ideal hydrogen production systems in many aspects, due to water management, dissolution, and high-cost problems, PEMWE stack studies are limited.^137^ The operating principle of a PEMWE stack system is classified into three different pressure ranges such as low-pressure, medium-pressure, and high-pressure stack systems. Today, commercial PEMWEs operate in the temperature range of 60 °C and a pressure range of 700 bar.^138,139^ This situation can vary between 1 bar in low-pressure systems and 700 bar in high-pressure systems. Different operating conditions for low-, medium-, and high-pressure PEMWE are given in Table 5.

**Table 5:** Comparison of PEMWEs Operating in Different Pressure Ranges

	Low Pressure	Medium Pressure	High Pressure	Ref.
Operating Pressure	1–20 bar	30–40 bar	40–700 bar	(140−143)
Operating Temperature	295–350 K	333–353 K	353 K	

**Figure 10:**
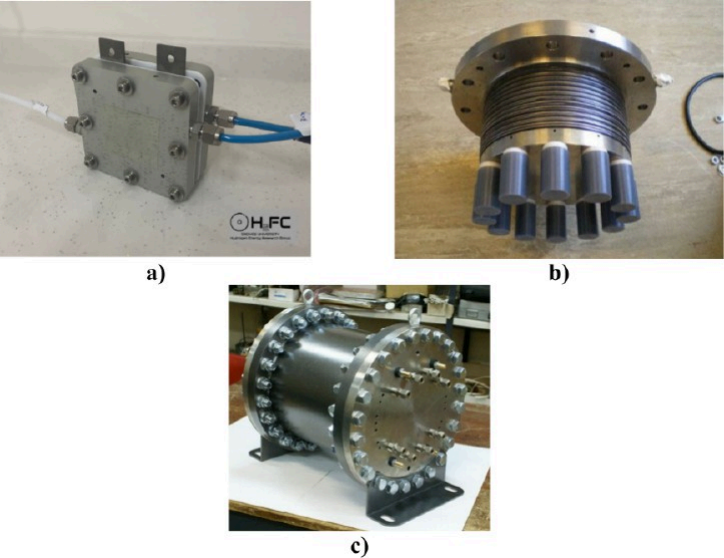
PEMWEs operate at different pressures: (a) low pressure (1 bar). Photograph courtesy of Murat Kıstı. Copyright 2022. (b) Medium pressure (30 bar) and (c) high pressure (130 bar). Reprinted using ref (144). Copyright 2011, Elsevier.

One of the parameters that allow operating at high pressures of PEMWEs is using membranes with low permeability. Although the studies conducted at high pressures have advantages compared to the atmospheric environment, there are also some disadvantages. PEMWEs operating under different pressures can be seen in Figure 10.

**Figure 11:**
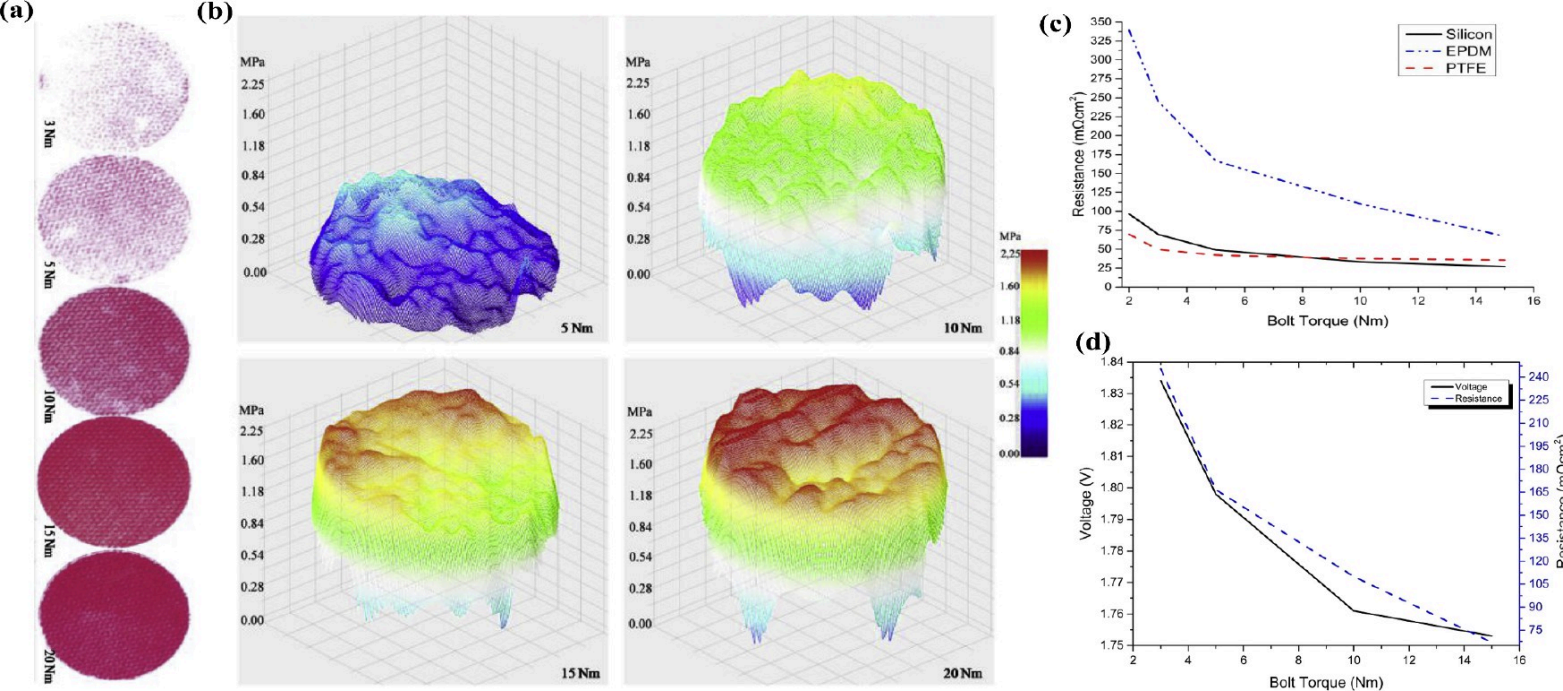
Pressure distribution of silicon gasket material: (a) Scanned pressure sensitive films, (b) 3D plot of pressure distribution profiles, (c) contact resistance measurements of each gasket material at 3, 5, 10, 15 N m clamping bolt torques, and (d) performance comparison at 0.5 A/cm^2^ and contact resistance versus bolt torque for the cell with EPDM gasket. Reprinted with permission from ref (159). Copyright 2015, Elsevier.

The gas removal performance of low-pressure systems, which are remarkably close to atmospheric pressure, is one factor limiting the PEMWE performance and efficiency. For this reason, the performance of medium-pressure PEMWE systems is considered more efficient.^10^ There are numerous studies on the effect of pressure on the system’s efficiency, hydrogen transition, and specific energy change. For example, Abdin et al.^145^ developed a numerical model to investigate the effect of some parameters on electrolyzer performance. Numerical modeling studies investigated the effects of cell performances at both low and high pressures and temperatures. According to the numerical modeling analysis results, they concluded that the efficiency of the electrolyzer is the best under elevated temperature and low-pressure conditions and attributed the elevated temperature of the cell to the increase in cell efficiency. Agate Martin et al.^146^ investigated the system’s hydrogen transfer and other parameters using a PEMWE with a Nafion 212 membrane. They examined the hydrogen transition analysis for PEMWE at two different operating pressures and kept the feedwater temperature at around 80 °C and the cathode pressure between 1 and 10 bar. They kept the anode pressure constant at 1 bar. They stated that the hydrogen transfer at ambient pressure increased due to the higher current density. Dinko Brezak et al.^31^ developed a model with variable operating conditions and compared this model with a commercial PEMWE. The PEMWE model used 15 cells with a total power of 15 kW. They noted that the pressure range in PEMWE operates about 1–20 bar, and the operating temperature is between 21 and 76 °C. They verified the accuracy of the modeling results by comparing them with the commercial PEMWE, and they concluded that the increase in electrolyzer efficiency at elevated temperatures can occur at low pressure. Joonas Koponen et al.^136^ stated that minimizing the amount of consumption in the specific energies of PEMWEs is due to the pressure selection and control at the hydrogen outlet. In their study, 5 kW of power is supplied for PEMWE from a solar power plant, and they used a differential pressure electrolyzer. In their experimental studies, they found that increasing the hydrogen output pressure from 20 to 40 bar does not cause a significant increase in energy consumption in PEMWE. However, they observed an increase in the consumption of specific energy when the pressure increased. Santarelli et al.^147^ designed a system to test electrolyzers’ mechanical strength and tightness from the cathode to the anode region. The anode side of low- or medium-pressure PEMWE stacks’ pressure is kept around 3 bar. On the cathode side is kept at 70 bar using a pressure valve, and hydrogen production occurs at this pressure value. Recently, studies have been carried out on high-pressure systems since the low output pressure of hydrogen poses a problem for the storage process.^148^ High pressure may become a good option for the cost-efficient production of hydrogen. However, increasing the pressure negatively affects the system, as it causes hydrogen to mix with oxygen in the stack system. It also allows water in the system to pass into the membrane, causing damage to the membrane and shortening its life. The highest pressure PEMWE is commercially around 700 bar.^138,149^ The smart hydrogen station developed by Japanese automotive manufacturer Honda is equipped with a high-discharge-pressure water electrolysis system. The smart hydrogen station with a pressure of 700 bar can produce and store hydrogen gas with electrical energy produced using renewable energy.^149^ It has been shown that PEMWE stacks operating at high pressures (10–150 bar) damage mass transfer under high pressure, and this situation should be controlled. Therefore, mass transportation can be increased by optimization of the cathode ionomer concentration, the membrane surface roughness, and the membrane thickness. Operation of PEMWEs under a high pressure can disrupt electrolyzer components. As a result of the studies, it has been determined that PEMWE components, such as membrane, BP, and GDL are significantly adversely affected in a high-pressure environment. Membrane types such as sulfonated poly(ether ether ketone), polybenzimidazole, and polysulfone, which have sufficient mechanical and chemical resistance under high pressure and temperature conditions, have been developed by researchers. PEMWE stacks at high pressure have two different operating types, such as balanced and differential modes. In differential pressure mode, the anode section operates at low or ambient pressure while the cathode section operates at high pressure. Proton exchange membranes allow differential pressure to be formed between the cathode and anode regions due to their structural properties.^148^ In balanced pressure mode, the hydraulic compression method is used between the anode and cathode regions. The compression pressure in the balanced pressure mode can also be adjusted during the electrolyzer stack operation. In high-pressure PEMWE systems can be hydrogen pressurized without emitting noise to the environment and requires minimal maintenance due to moving mechanical parts.^150,151^ A sizable differential pressure occurs between the anode and cathode parts of the PEMWE. The studies show that the pressure difference between the two parts is up to 350 bar. In differential pressure mode, it can eliminate the hazards associated with the transport of pressurized oxygen and the possibility of self-ignition of metals. The purpose of the pressure gradient is to partially return the oxygen accumulated in the membrane to the anode, thus minimizing the passage of oxygen.^152^ A PEMWE with differential pressure modes can produce hydrogen from 30 to 40 bar. Bensmann et al.^153^ theoretically compared differential and balanced pressure modes for high-pressure hydrogen production using water electrolysis. The differential pressure mode performed better at hydrogen pressures of up to 40 bar. It is determined that the energy required for hydrogen production increased at high pressures in the balanced high-pressure mode.^148^ Moreover, promising experimental investigations on the use of expanded metals as flow field plates or porous media have been conducted, encompassing both single-cell and stack applications. Since these metals can be defined as porous media, the sealing of flow field areas is important, especially in terms of crossflows. Sealing elements must be able to withstand the pressures present within the electrolyzer stack and they provide insulation and sealing of the anode and cathode compartments, preventing gas passage in PEMWEs.^154^ The choice of material and design should consider the pressure differentials across the seals during operation. Proper sealing is essential to prevent unwanted cross-mixing of reactants and products between different channels, ensuring efficient and controlled reactions within the fuel cell. Effective sealing is vital for maintaining the integrity of the electrochemical processes and optimizing the overall performance of the fuel cell system.^155^ PEMWEs often use a variety of sealing materials to ensure efficient and safe operation of the system. These materials must be compatible with the electrolyte, withstand operating conditions, and provide a tight seal to prevent leaks. To keep the overall performance of the PEMWE system at an optimum level, the sealing materials need to be carefully selected based on factors such as compatibility with the electrolyte, operating temperature, and pressure, durability, and cost-effectiveness. Various polymeric materials may be used for sealing applications in PEMWEs, depending on the specific requirements of the system. These materials include elastomers such as EPDM (ethylenepropylene diene monomer) or certain fluoropolymers other than Perfluoroelastomers. PTFE is widely used as a sealing material in PEMWE systems due to its high chemical resistance and low coefficient of friction. PTFE exhibits excellent resistance to a wide range of chemicals, including strong acids and bases, which are commonly encountered in the operation of PEMWE systems. Moreover, PTFE has a very low coefficient of friction, making it an ideal material for sealing applications.^156^ In some cases, especially in high-pressure or high-temperature applications, metal gaskets made of stainless steel or nickel alloy materials are used in PEMWEs as they provide excellent mechanical strength and durability. On the other hand, silicone rubber, which is known for its flexibility, thermal stability, and oxidation resistance, used for sealing applications in PEMWEs can be utilized especially in less critical areas where exposure to high temperatures and aggressive chemicals is minimal.^157,158^ For example, Selamet et al.^159^ have examined the effects of different sealing materials in metal meshes for PEMWEs. They measured the effect of clamping pressure on cell performance and contact resistance for each gasket material. Moreover, they have determined bolt torque values suitable for each situation for the PEMWE. In their analysis results, they commented that the contact pressure in the flow area is largely based on the sealing materials, as seen in Figure 11. In particular, with regard to the sealing materials used in electrolyzers, attention should be paid to the material strength and mechanical properties during operation in terms of long-term stability. The selection of seals is often an important part of the overall system design process, and seal materials may vary depending on the specific electrolyzer design, operating conditions, and application requirements. Sealing materials must be compatible with the electrolyte and other chemicals present in the system. Moreover, sealing materials used must be suitable for the operating requirements of the cell and must also be cost-effective.^157,160^

## 4 Recent Manufacturing Technologies for PEMWE Stacks

**Figure 12:**
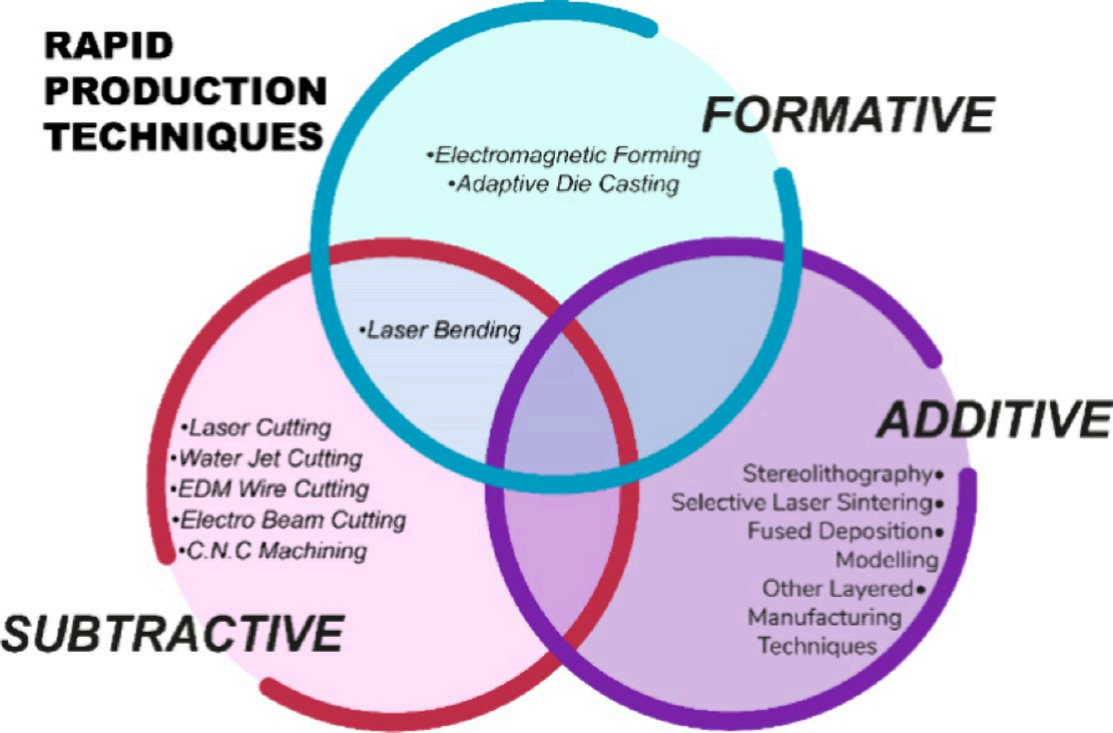
Rapid production techniques. Reproduced using ref (161). Copyright 1999 Springer Nature

Significant improvements have been made in the production and processing methods necessary to develop industry-oriented innovative models in the last ten years. The manufacturing method, widely used in industry, can come to the fore in traditional manufacturing methods, such as machining and chipless machining. Moreover, manufacturing methods such as laser, plasma, and pressurized water jets are widely used in the industry, and these techniques can be classified as subtractive, additive, or formative. Current production techniques may be included in one of these categories or may follow a hybrid path by inclusion in more than one category. Figure 12 shows a diagram representing rapid production techniques.

**Figure 13:**
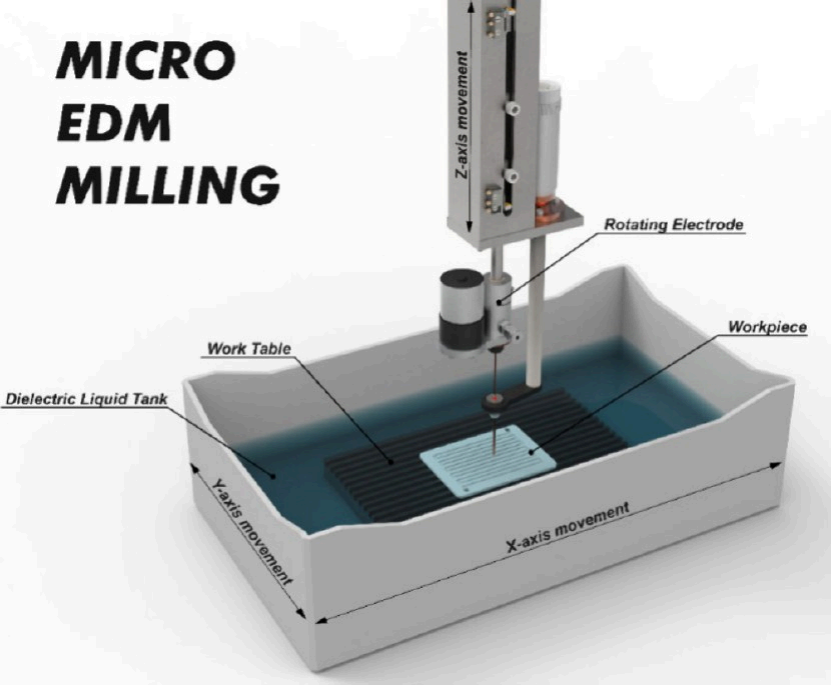
Schematic of micro EDM milling method. Photograph courtesy of Yakup Ogün Süzen. Copyright 2023.

Prototyping and model-making are essential steps in designing a product, and they can help them conceptualize, develop, and test design. In the mid-1970s, a soft prototype’s material and other properties were simulated using its 3D curves and modeling surfaces in a virtual environment. In the early 1980s, with the growth and development of computer-aided design (CAD) and computer-aided manufacturing (CAM) technologies, producing CNC machining by defining parameters such as edge and surface information in the model became possible. BPs are one of the crucial components in PEMWEs, and the cells and stacks must have the necessary mechanical strength to withstand the compression load and provide structural support to the cell. Therefore, cell strength and low ICR are among the critical properties of BPs to improve electrolysis performance.^162^ Although graphite, the most widely used in producing BPs, has high electrical conductivity and corrosion resistance, metallic BPs have been used instead of graphite sheets due to their poor machinability, fragility caused by their microstructure, and lack of mechanical strength. However, due to the low corrosion resistance of metallic BPs in the PEMWE environment, the coating process may be required for BPs.^163^ By using composite or metallic plates instead of graphite sheets, it has been predicted that plate costs in a stack can be reduced from 60% to 15–29%.^164^ In order to enhance the long-term competitiveness of fuel cells, it is necessary to lower the cost of the MEA. As depicted in Figure 8, the high cost of fuel cells is primarily due to the expensive PGM catalysts, which are material costs that do not decrease with increased production. Therefore, it is crucial to reduce the amount of PGM catalyst used while ensuring durability. Although many types of CNC machining are used in the production and processing of BPs, the CNC micro electrical discharge machining milling (micro EDM milling) method is generally preferred for processing flow channels due to its high precision and flexible machining advantages. Figure 13 shows a schematic of the EDM milling method.

**Figure 14:**
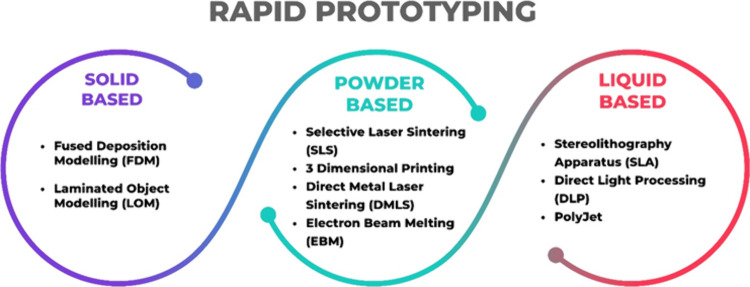
Classification of the RP methods.

This method is known as a thermal process that uses electrical discharges to process electrically conductive materials. In this process, the material is formed between two electrodes (tool and workpiece) immersed in the dielectric fluid, and it is removed in the form of small particles called chips (debris) by the succession of repeated controlled sparks.^165^ For example, Hung et al.^166^ used the microimmersion erosion (Die-sinking EDM) method. They asserted a faster technique for the fabrication of BPs than other fabrication methods for the three-pass serpentine flow channel structure on SS316L. Hung et al.^167^ studied the performance of PEM fuel cells using the micro EDM milling method for high aspect ratio microflow channels in metallic BPs. They observed that a high aspect ratio metallic BP structure with the micro EDM method could provide an efficient approach to improve cell performance. In addition, they observed that the power density of metallic BP with a channel aspect ratio of 1.2 is higher than that of the 0.6 aspect ratio. So, the cell performance could be increased by increasing the aspect ratio of the flow channel. Simaafrookhteh et al.^168^ conducted the production of thermoset-based composite BPs using the hot compression molding process. However, they observed that the surface appearance may deteriorate during the process and cause internal defects. For this reason, they fixed the mold surface using a suitable temperature and press machine, and they also stated that graphite-intercalated compound (GIC-graphite-intercalated compound) should be used to obtain a high-quality surface on the parts. Rapid prototyping (RP) technologies have developed in recent years due to the limitations and costs of traditional manufacturing techniques. RP technologies have a prominent place, because they can produce complex designs in a brief time. One of the biggest challenges in commercializing PEMFC and PEMWE technologies is new and innovative ways to manufacture different components that can be mass-produced at low machining costs. The three-dimensional (3D) printing method, called AM, has an essential place in producing complex parts that cannot be produced using traditional production methods as well as metallic or polymer parts. It can be produced that a wide variety of materials using the AM method, including ceramics, glass, metals, polymers, and composite materials.^27,169,170^ By using the AM method, it is possible to significantly shorten the production of fuel cell prototypes in terms of both time and cost. A diagram showing the rapid prototyping methods is given in Figure 14.

**Figure 15:**
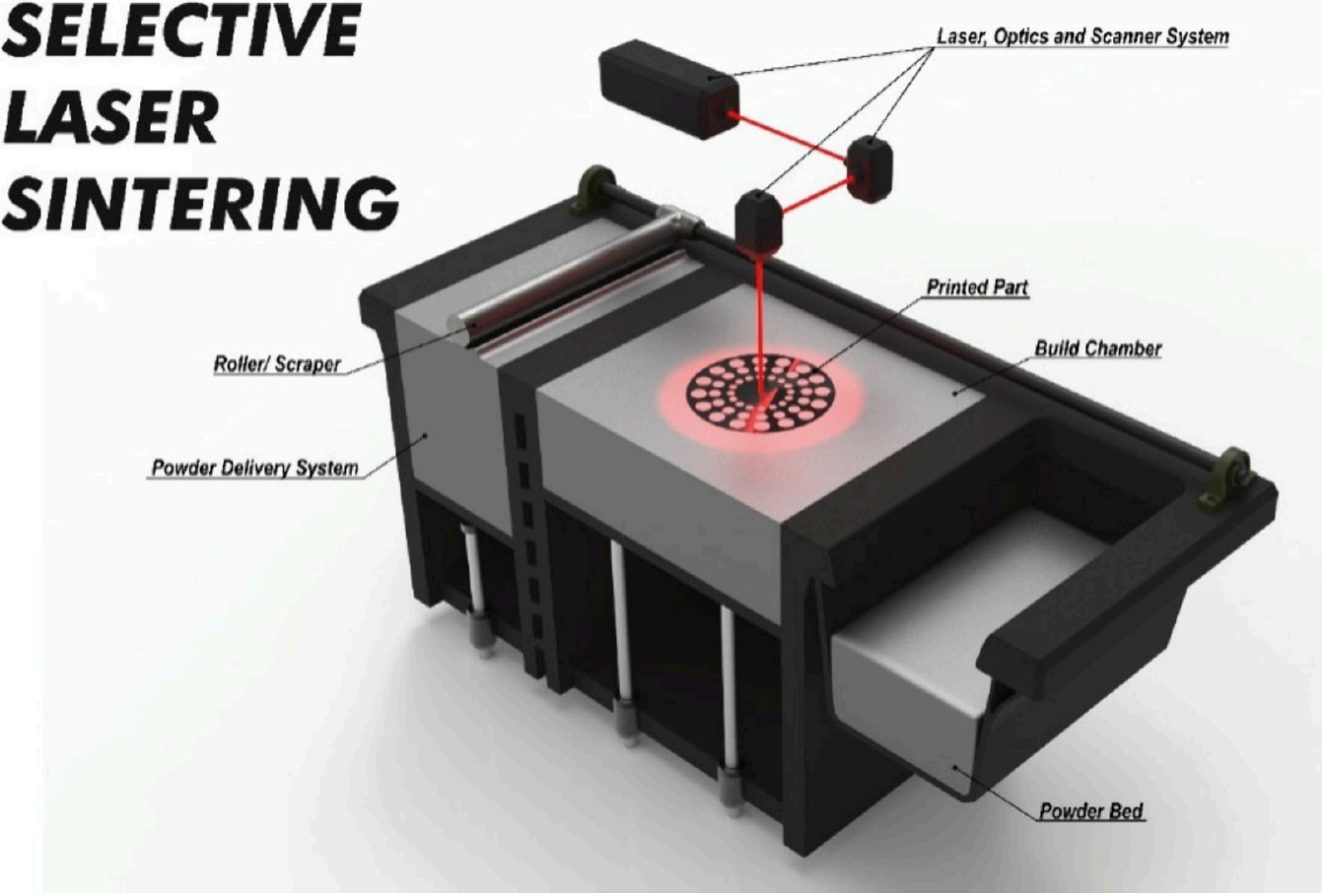
Working principle of the SLS method. Photograph courtesy of Yakup Ogün Süzen. Copyright 2023.

AM technologies, such as selective laser sintering (SLS), direct metal laser sintering, stereolithography (SLA), or fused deposition modeling (FDM) can be used to manufacture parts in a particularly planar configuration. Figure 15 shows a working schematic of the SLS method.

Mo et al.^171^ achieved an 8% improvement in operational efficiency over conventional woven and sintered GDLs by using electron beam melting (EBM) to create a Ti6Al4 V GDL with variable parameters. This improvement can be attributed to the interconnected circulatory system made possible by 3D printing, which reduces ohmic losses by providing precise control over the pore size, shape, and distribution. Because of a shielding layer of titanium oxide formed during passivation, the inexpensive 3D printed Ti6Al4 V GDL also demonstrated exceptional corrosion resistance. Future applications of hybrid and multimaterial additive manufacturing in fully integrated fuel cell systems are suggested in this research. Over the past decade, many researchers have used elementary flow field geometries in fuel cells due to the limitations of conventional fabrication techniques. 3D printing is an excellent rapid prototyping method for BPs prototyping to experiment on new flow field designs. Jin et al.^172^ investigated the use of Powder Bed Fusion (PBF) 3D printing to manufacture an SS316L BP with rectangular microchannels and micro ribs in order to improve fuel cell performance. They designed a bipolar plate with a triple serpentine flow field and 300 μm channel width and rib, resulting in a current density of 1.2052 A/cm^2^ at 0.6 V. This is a 52.8% increase compared to plates with 940 μm channels and a 24.9% increase over graphite plates. The findings point out the potential of PBF 3D printing to improve fuel cell performance by allowing fine control over the microchannel architecture. Flow field designs for BPs are produced using PolyJet, SLA, and laser cutter technologies, and pressure drop and velocity profiles are measured for each plate.^173^ For example, Piri et al.^173^ investigated the applicability of BPs with different 3D printing methods. They stated that the PolyJet method is unsuitable for small-sized 3D printing. There are problems with the PolyJet 3D printing process, such as being prone to deformation and damage. In some cases, it is observed that the channel walls are damaged after washing the plates, and the material is deformed when it is exposed to water. They reported that 3D-printed channels prepared using a laser jet and SLA are almost like one another and the surface roughness of the channels is high. Therefore, the channel pressure drop increased and disrupted the flow distribution. They also noted that BPs prepared using the laser jet method cost less (about one-third) than 3D-printed BPs prepared with the SLA method. It is developed by the SLA method 3D System company for rapid prototyping purposes.^174^ The photopolymerization method (SLA) involves curing a photosensitive monomer resin using a scanning laser or UV radiation and transferring the photo resin liquid to a cross-linking.^175,176^ In addition to essential features such as high precision, mechanical strength, and smoothness with the SLA method, it provides high-quality 3D printing with cost-effective and short prototyping time.^173^ Because BPs have an essential place in the production costs of electrolyzers such as PEM and AEM, the material type, design, and production method can affect the performance and durability of the electrolyzer stack.^177^ In conclusion, CNC techniques for prototyping are common in BP production. Sheet metal forming is also used for mass production. However, using rapid prototyping manufacturing techniques can offer numerous advantages, such as low stack cost and high design flexibility, which may not be possible with traditional manufacturing methods. Although 3D printing is highly researched, it is not a cure-all technology. Rapid heating and cooling during some additive manufacturing processes can lead to microcracks, residual stresses, and porosity in 3D printed parts. These factors can impact the performance of components, potentially causing leaks in fuel cell systems and making it difficult to ensure product repeatability under the same manufacturing conditions.^178,179^ Moreover, overall, a more efficient and cost-effective development process in PEMWE, the SLA has a promising method among rapid production techniques.

## 5 PEMWE Applications and Recent Commercial Situation

The industrialization of hydrogen production technology occurred between the 1920s and 1970s, particularly for applications such as petroleum refining. From the 1970s to the present, technological developments in space exploration and military applications have facilitated the advancement of PEMWEs. Electrolyzer manufacturers around the world made significant efforts, and the Aswan company installed a hydrogen generation facility with a capacity of 162 MW, producing 32,400 m^3^ of hydrogen using 144 electrolyzers by the late 1980s. In 1900, water electrolysis was still in its early stages commercially. Two decades later, large-scale electrolyzer plants with a capacity of 100 MW were improved in Canada for fertilizer production.^180−182^ These developments have facilitated the implementation of PEMWEs in renewable energy systems for the production of green hydrogen in solar- or wind-powered electrolyzers, marking a significant advancement in the field of sustainable energy. On the other hand, the factors that contribute the most to the material cost of PEMWE stacks for performance enhancement are the coatings (Pt, Ir, and Au, etc.), the metallic powders required for the PTL anode (SS316L and Ti), and the Nafion membrane structures. The advanced PEMWE designs for 2030 depend on a current density of 3.5 A/cm^2^.^183^ To achieve this high current density, expected improvements in cell design such as thinner membranes, reduced PGM loadings, and cheaper materials are incorporated into the basic electrochemical model as outlined in IRENA’s Green Hydrogen Cost Reduction report. These innovations aim to achieve high-efficiency, high-output PEMWEs capable of producing green hydrogen with a lower Levelized Cost of Hydrogen (LCOH), supporting the widespread adoption of hydrogen technologies by 2030.^184^Table 6 provides a summary of the stack properties for the baseline and advanced designs for PEMWE.

**Table 6:** Comparison of PEMWE Stack Properties of Basic and Advanced Designs^185^

	Baseline Properties for 2020	Advances Properties for 2030
Stack power	0.67 MW	9.75 MW
Number of cells	150	310
Cell Area	0.10 m^2^	0.50 m^2^
Power density	4.5 W·cm^–2^	6.3 W·cm^–2^
Current density	2 A·cm^–2^	3.5 A·cm^–2^
Pressure	20 bar	30 bar
Temperature	55 °C	70 °C
Voltage	2 V	1.8 V

**Figure 16:**
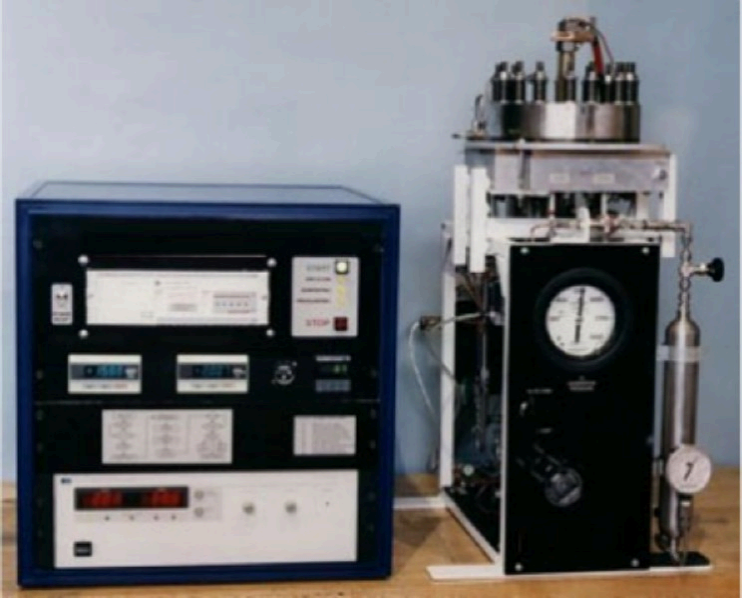
Representative photograph of the OBOGS/PEMWE system. Reprinted using ref (190). Photograph by Fred Mitlitsky. Copyright 1999.

PEMWEs have many advantages, such as operating at a higher current density above 2 A cm^–2^, fast dynamic operation, high gas purity, and high voltage efficiency.^13,89,186,187^ Although the temperature and pressure are among the main factors affecting the working environment of PEMWEs, water management plays a crucial role in the PEMWE system. Therefore, to make hydrogen production more efficient by using PEMWEs and to offer innovative prototypes, studies have been conducted on the development of components and integration into the PEMWE system. According to a report by the National Renewable Energy Laboratory (NREL),^188^ they discussed the critical aspects of commercializing Giner ELX’s PEMWE stacks at MW levels. They first formed a multicellular stack of 1 MW with an active surface area of 1250 cm^2^. They determined the number of cells based on their power capabilities at NREL’s Energy Systems Integration Facility (ESIF) test field. The prepared electrolyzer stack consists of 29 cells and can operate under a pressure of 40 bar. Serna et al.^189^ proposed designing an off-grid offshore electrolysis plant powered by wave energy. After the design proposal of the facility, a desalination system for PEMWE, and hydrogen compression, the researchers proposed a controller and provided some guidelines for sizing the facility for specific locations. They noted that this sizing depends on a multicomponent model-based simulation that considers buoy-measured data at the determined location and the effect of measured sea conditions on hydrogen production. Barbir et al.^5^ discussed several applications, which is including off-grid and off-grid hydrogen production, the use of an electrolyzer system, and both grid-connected and off-grid systems via the fuel cell, where the electrolytically produced hydrogen is stored and then converted back into electricity when needed. Moreover, they examined parameters suitable for using PEMWE in renewable energy systems, such as electrolyzer sizing, outlet pressure, oxygen production, water consumption, and electrolyzer efficiency. For the PEMWE system, they used HOGEN 40, an industrial electrolyzer manufactured by Proton Energy Systems. Mitlitsky et al.^190^ investigated prototyped PEMWEs suitable for product development and application guidelines for high-pressure PEMWEs. They also discussed aerospace-related PEMWE stacks, which were already developed up to 1999. Figure 16 shows the On-Board Oxygen Generating System (OBOGS) system.

**Figure 17:**
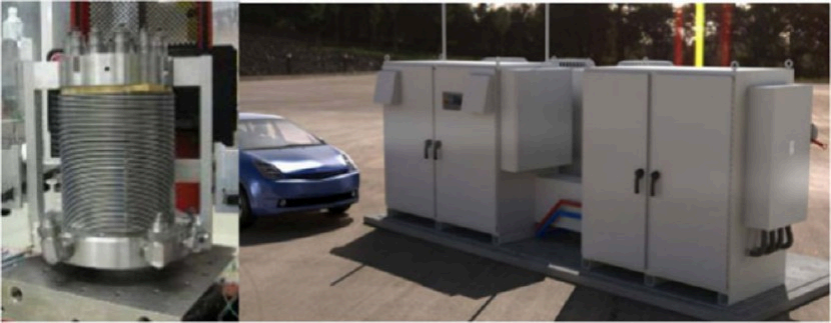
PEMWE and system concept with a production capacity of 65 H_2_ kg/day; reprinted using ref (6). Photograph courtesy of Katherine E. Ayers. Copyright 2010.

Grigoriev et al.^144^ studied the safety aspects related to the operation of PEMWEs (hydrogen production capacity:1 N m^3^/h and working pressure: 130 bar). They designed a PEMWE operating at temperatures up to 90 °C and 0–130 bar as a part of the GenHyPEM project, a research program on high-pressure PEMWE supported by the European Commission. Moreover, they have identified the hydrogen concentration and membrane crossover effects in oxygen gas production as the most critical risks. According to the results, the oxygen concentration in hydrogen at 130 bar could reach up to 2.66% by volume and is outside the flammability limit (3.9–95.8% by volume) for hydrogen–oxygen mixtures. However, they noted that safety measures to prevent explosion hazards. Ayers et al.^6^ studied hydrogen production systems with various PEMWEs in the United States until 2012. According to their research, hydrogen-producing stations with a typical PEMWE capacity is 12 kg/day H_2_, and hydrogen is produced between 15.8 and 29.6 bar pressure value. They also reported that the hydrogen produced at these stations could be compressed up to 689.4 bar for vehicle use.^191^ Therefore, a much larger hydrogen generation station has been initiated in the USA for bus refueling and small vehicle fleets. In this way, the hydrogen production station with a capacity of 65 kg/day H_2_ (30 N m^3^ H_2_/h) has been serving successfully since 2010. Figure 17 shows a photograph of the stack and system concept.

As seen in Figure 17 it is stated that the hydrogen output pressure of the PEMWE system is optimized up to 800 psi. In addition, high-pressure PEMWE designs are required for this type of high-pressure hydrogen production. The high-pressure hydrogen production process is more efficient than the low-pressure postelectrolysis process.^151^ PEMWEs that can produce hydrogen at high pressure are frequently preferred in applications, and some companies develop commercial electrolyzers in addition to all these PEMWE prototypes. The manufacturers that perform the mass production of PEMWE and their characteristic properties are listed in Table 7.

**Table 7:** Some Commercial PEMWE-Producing Companies and Their Details

Manufacturer	Model	Release Year	Operating Pressure (bar)	Hydrogen Flow Rate (N m^3^·h^–1^)	Energy Consumption (kWh·Nm^–3^·H_2_)	Power	Refs
H-TEC Systems	ME450	2019	30	210	4.7 (by stack)	1 MW	(192)
	HCS			420		2 MW	
	MHP			2130		10 MW	
NEL Hydrogen	C10	2024	30	10	6.2 (by system)	N/A	(193−197)
	C20			20	6.0 (by system)		
	C30			30	5.8 (by system)		
	H2	2021	15	2	7.3 (by system)		
	H4			4	7.0 (by system)		
	H6			6	6.8 (by system)		
	S10	2019	13.8	0.27	6.1 (by system)		
	S20			0.53			
	S40			1.05			
	MC100	2019	30	103	4.53 (by stack)	0.5 MW	
	MC200			207		1 MW	
	MC250			246		1.25 MW	
	MC400			413		2 MW	
	MC500			492		2.5 MW	
Elogen	E500	2023	30	500	4.4 (by stack)	2.5 MW	(198)
	E1000			1000		5 MW	
Hydrogenics	HyLYZER 200	2019	30	200	4.8 (by stack)	1 MW	(199, 200)
	HyLYZER 250		30	250		1.25 MW	
	HyLYZER 300		30	300		1.5 MW	
	HyLYZER 400		30	400		2 MW	
	HyLYZER 500		30	500		2.5 MW	
	HyLYZER 1000		30	1000		5 MW	
	HyLYZER 4000		30	4000		20 MW	
	HyLYZER 5000		30	5000		25 MW	
Areva H2Gen	Elyte	2019	35	10–200	4.7–5.3 (by stack)	80–1600 kVA	(201)
Siemens	SILYZER 200	2016	35	225	N/A	1.25 MW	(202)
ITM Power	Trident	2024	N/A				(203)
	Neptune						
	Poseidon						
Humble Hydrogen	H2 series	2021	40	200	N/A	1 MW	(204)

**Figure 18:**
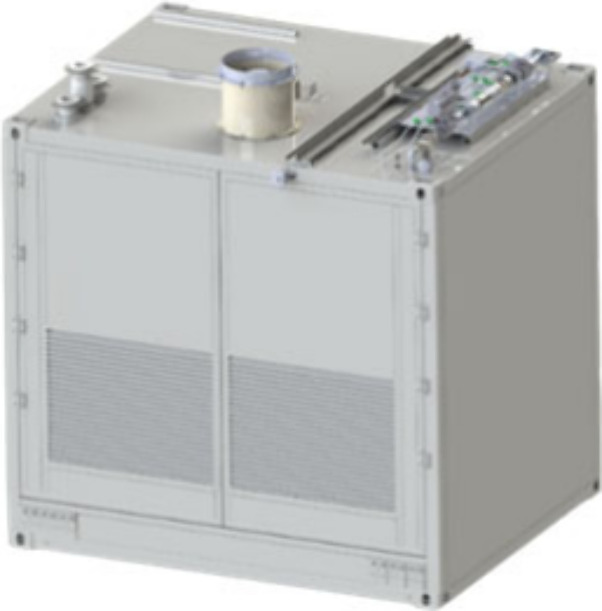
3MEP CUBE PEMWE model developed by ITM Power. Reprinted using ref (206). Photograph courtesy of ITM. Copyright 2021.

As seen in Table 7, it is seen that commercial PEMWEs have reached a power level of MW. In addition, the highest pressure of 30 bar could be reached in commercial PEMWEs. There has been a noticeable reduction in the energy required to produce a specific quantity of hydrogen as newer models of commercial products are introduced. For instance, when comparing models from NEL Hydrogen released in 2021 with those from 2024, it is evident that despite an increase in operating pressure, there’s a decline in system-based energy consumption. Furthermore, the MC500 model by NEL Hydrogen, introduced in 2019, boasted a hydrogen flow rate of 492 N·m^3^·h^–1^. In contrast, the E500 model by Elogen, released in 2023, achieved a hydrogen flow rate of 500 N·m^3^·h^–1^ with a lower energy consumption at the same power depth. These instances compellingly illustrate the ongoing advancement of PEMWE technology. Apart from the mass production models (Table 6), Plug Power installed hydrogen production systems that can be integrated with renewable systems from 1 to 5 MW power values according to a user’s request.^205^ Moreover, thanks to the 3MEP CUBE model developed by ITM Power, embedded system PEMWEs are prepared, and the 3MEP CUBE model PEMWE can be seen in Figure 18.

**Figure 19:**
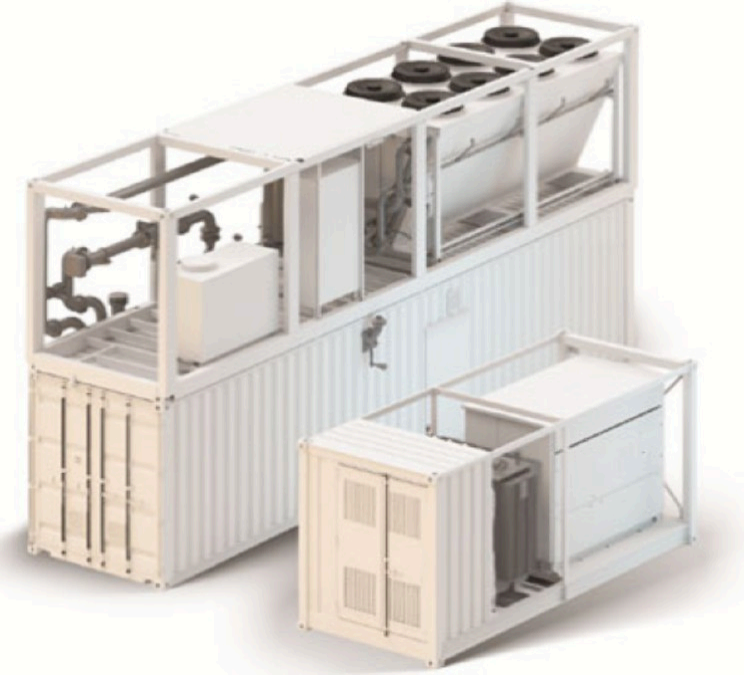
NEL hydrogen M series PEMWE system. Reprinted using ref (194). Photograph courtesy of NEL Hydrogen. Copyright 2023.

As can be seen in Figure 18, the 3MEP CUBE model is a commercialized PEMWE product that can operate at a pressure of 30 bar and has a maximum hydrogen production capacity of 36 kg/h. NEL hydrogen company has developed a container-sized PEM-type electrolyzer that can produce hydrogen between 246 and 492 N·m^3^·h^–1^ hydrogen flow rate. Figure 19 shows the M series electrolyzer system of the NEL hydrogen company.

**Figure 20:**
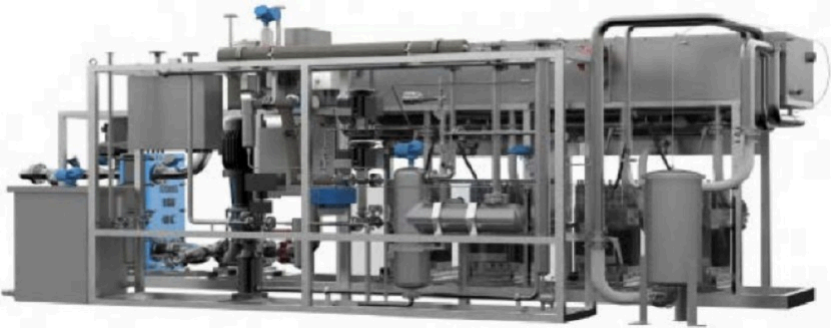
Proton M series M100 PEMWE system. Reprinted using ref (195). Photograph courtesy of NEL Hydrogen. Copyright 2023.

The M series PEMWE shown in Figure 19 has a hydrogen production capacity of 531 kg/24 h, and its delivery pressure is 30 bar. Moreover, the power consumption of the PEMWE stack is 4.5 kWh/Nm^3^. The Proton company produced M100, M200, and M400 products under the name of M series. Figure 20 shows the Proton M100 PEMWE product.

The M100 PEMWE, shown in Figure 20, can produce 225 kg/24 h hydrogen with an output pressure of 30 bar. The PEMWE stack has a power consumption of 0.51 MW, while the entire system consumes 0.55 MW. NEL hydrogen and Proton container-type high-flow PEMWE systems have a start-up time of less than 5 min. Considering all these situations, there are companies such as Bloom Energy, Thyssenkrupp, Cummins New Power, Bataryasan, and Aspilsan continue to develop PEMWE stacks. Both companies stated that they turnkey PEMWE systems to their users and that they could easily take them into operation. In addition to all these systems, PERIC Hydrogen Technologies (China), CNNE Technology (China), Suzhou Green Hydrogen Energy (China), Areva H2Gen (France), Elogen (France), NedStack (Netherlands), Bloom Energy (United States), Cummins New Power (United States), Thyssenkrupp (Germany), Aspilsan (Türkiye) and Bataryasan (Türkiye) companies such as PEMWE stacks continue to develop and produce. In this section, commercially available PEMWE systems are included. These products for applying PEMWE systems show that the production of hydrogen in clean ways is developing in the sector. Users who need hydrogen industrially can meet their needs with PEMWE systems. Development studies continue to reduce the cost of PEMWE systems, and their energy need from renewable energy systems.

## 6 Commercialization Issues of PEMWE

In recent years, hydrogen production integrating new and clean energy sources, such as solar and wind, has led to the development of MW-scale systems in many applications. PEMWEs have significant advantages over the other electrolysis technologies, such as a higher rate of hydrogen production, scalability, grid balancing capability, high load flexibility, operation at higher current densities (up to 10 A·cm^–2^), and high hydrogen purity (up to 99.9999%).^207,208^ Moreover, due to their control capabilities, such as fast commissioning and fast response to load, PEMWEs can be integrated into smart homes, e-mobility, and e-fuel systems.^209,210^ Today, technology is available for commercial products on the MW scale. However, several improvements are needed to reduce the cost of electrolytic hydrogen to 5 €.kg^–1^ levels for various applications in European countries. Therefore, one of the main challenges to overcome is replacing electrocatalysts containing Pt group metals with non-noble electrocatalysts or using them as alloys (containing less noble metal content).^211^ During the electrolysis process, alternatives for PGM are required to ensure sensitivity to low amounts of minerals and impurities in the water. PEMWE requires specific water quality parameters for optimal performance and durability. The American Society for Testing and Materials (ASTM) has established strict requirements for commercial PEMWEs, recommending Type I deionized water with less than 50 ppb of total organic carbon, a resistivity exceeding 1 MΩ-cm, and sodium and chloride content below 5 μg L^–1^.^212^ Several water quality parameters significantly impact the PEMWE efficiency. The pH of the electrolyte influences both hydrogen production and energy consumption, where a lower pH reduces the OER potential, resulting in decreased energy consumption. However, this benefit must be balanced against potential membrane degradation at extremely low pH levels.^213^ Conductivity also plays a crucial role, as higher conductivity reduces the overall potential and energy requirements, although excessive conductivity can damage the membrane. Additionally, there is an asymmetric and pH-dependent distribution of reactive excess overvoltage between HER and OER. Total dissolved solids (TDS) present an interesting case, with some studies showing improved production at TDS levels between 0 and 2000 ppm, while others demonstrate that certain impurities, such as calcium and magnesium ions found in soft river water, can significantly degrade electrolyzer performance and reduce cell life.^214^ This requirement for high-purity water presents a significant economic challenge, as most available water sources require additional purification steps, adding to overall system costs. The optimization of these parameters, pH, TDS, and conductivity, is therefore critical for achieving enhanced PEM electrolyzer performance while maintaining system longevity. However, in practical applications, impurities may still enter an electrolyzer despite these requirements.^215^Table 8 provides a comparative analysis of ionic impurity, and water requirements in three different water types: seawater, tap water, and ASTM Type II water (1 MΩ cm). Both tap water and seawater, which are commonly used feedstocks for water electrolysis, must undergo purification processes to meet the ASTM Type II water specifications before they can be used in PEMWE.

**Table 8:** Comparison of Water Requirements in PEMWE

Ion	Seawater Conc. (mg L^–1^)^216^	Tap Water Conc. (mg L^–1^)^217^	ASTM Type II Water (μg L^–1^)^212^
Na^+^	11000	62	≤0.005
Ca^2+^	400	51	-
Mg^2+^	130	7	-
Al^3+^	-	0.004	-
K^+^	400	-	-
Cl^–^	200	79	≤0.005
Br^–^	10	-	-
F^–^	1	-	-
HCO_3_^–^	110	158	-
CO_3_^–^	20	-	-
SO_4_^2–^	2800	47	-
NO_3_^–^	-	0.82	-

As seen in Table 8, ASTM Type II water contains Na^+^ and Cl^–^ ions (5 μg L^–1^ each), leading to conductivity of 1.0 μ S cm^–1^. In a 1 MW PEMWE stack operating for 10 years, this results in 40 g of Na^+^ exposure, affecting one-third of the membrane capacity. As water is consumed but impurities accumulate, their concentration increases significantly, necessitating ion exchange resins in the recirculation loop.

In addition, there is a need for cheaper catalyst materials due to the high cost of IrO_2_ and RuO_2_-based catalysts with high kinetic activity used on the anode side of PEMWEs.^218^ Although the IrO_2_ loading on the anode side of PEMWE can be reduced to 0.5 mgcm^–2^ levels, the MEA activity and resistance may be reduced against high loading amounts. The increase in loading rate provides better durability but causes prohibitive costs. Therefore, an optimization must be made between the cost and durability of the catalysts.^219,220^ Two essential options to reduce the cost of PGMs are optimizing the loading amount and replacing these metals with alternative catalyst materials. Technological advances in fuel cell technology have led to the development of carbon-supported Pt nanoparticles. These carbon-supported Pt nanoparticles can be used directly on the cathode side of PEMWE for HER.^221^ The gradual transition is foreseen by 2030 by integrating renewable energy sources of PEM fuel cell technology.^222^Table 9 presents the United States Department of Energy (DOE) technical targets (mainly for cost situations) to achieve for PEMWEs.^223^ When Table 7 is examined, there is a low-cost hydrogen production target of 2 $/kg H_2_ by 2026 and 1 $/kg H_2_ by 2031. It is seen that these targets depend on many combinations of energy efficiency, lifetime, and capital cost.

**Table 9:** Technical Targets for PEMWE Stacks and Systems for H_2_ Production^223^

Properties	Units	2022 Status	2026 Targets	Ultimate Targets
PEMWE Stacks Status				
Total PGM Catalysts Content (both anode and cathode electrode)	Mg·cm^–2^	3.0	0.5	0.125
	g·kW^–1^	0.8	0.1	0.03
Performance		2.0 A·cm^–2^@1.9 V/cell	3.0 A·cm^–2^@1.8 V/cell	3.0 A·cm^–2^@1.6 V/cell
Electrical Efficiency	kWh·kg^–1^ H_2_ (LHV%)	51 (65%)	48 (69%)	43 (77%)
Average Degradation Rate	mV·kh^–1^ (%/1000 h)	4.8 (0.25)	2.3 (0.13)	2.0 (0.13)
Lifetime	h	40,000	80,000	80,000
Capital Cost	$·kW^–1^	450	100	50
PEMWE System Status				
Energy Efficiency	kWh·kg^–1^ H_2_ (LHV%)	55 (61%)	51 (65%)	46 (72%)
Uninstalled Capital Cost	$·kW^–1^	1,000	250	150
H_2_ Production Cost	$·kg^–1^·H_2_	>3	2.00	1.00

The performance, durability, and capital cost targets of PEMWEs must be compared in the same stack or system to achieve the H_2_ production cost targets. Therefore, these targets have been given in stacks and systems related to commercial scales. In particular, Ir metal as the anode catalyst accounts for most of the PGM content in PEMWE. On the other hand, Pt is used as the cathode catalyst. Reducing PGM catalyst loading in stacks and systems is essential to reduce cost. However, performance and durability or stability targets must be met to achieve this state.

## 7 Perspective on Future Research Directions in PEMWE Technology

The future development of PEMWEs hinges on addressing critical technological, economic, and environmental challenges. Advancing this technology to meet the increasing demand for green hydrogen production requires an interdisciplinary approach that integrates innovations in materials, manufacturing, and system design with comprehensive cost and environmental assessments. This section outlines key areas for future research that could significantly enhance the efficiency, scalability, and affordability of the PEMWE systems.

A primary focus of future research should be material innovations to overcome the limitations of the current PEMWE components. Catalysts, for instance, are a significant cost driver due to the reliance on precious metals, such as iridium and platinum. Developing earth-abundant alternatives, hybrid composites, or atomically dispersed catalysts with similar catalytic activities and stabilities is essential. Additionally, recyclable catalyst materials should be explored to lower the long-term operational costs and environmental impacts. Membrane technology also requires substantial advancements. New materials that combine high proton conductivity with exceptional thermal and mechanical stability under high-pressure and high-temperature conditions could redefine the system performance. Innovations in ionomer structures and hybrid polymer composites, particularly those with nanostructured reinforcements, are promising avenues. GDLs, another vital component, should be made from corrosion-resistant yet cost-effective alternatives to titanium such as stainless steel or composite structures with advanced protective coatings.

Another promising area lies in manufacturing advancements to address cost and scalability challenges. AM, including techniques such as SLS and EBM, has demonstrated potential in creating components with optimized geometries for better mass and heat transfer. These techniques can enable the production of lightweight, high-performance BPs and gas diffusion media, contributing to reduced system costs. Moreover, new surface engineering and coating technologies could enhance the corrosion resistance and electrical conductivity of metallic components. For instance, developing multifunctional coatings with self-healing properties could significantly improve the durability and lifespan of PEMWE systems.

System integration and scale-up represent other critical research direction. Designing stack configurations that ensure uniform current distribution, efficient water management, and reduced hydrogen crossover is imperative for large-scale deployment. Future designs should consider modular and compact stack systems that can be easily integrated into renewable energy infrastructures. Moreover, intelligent control systems equipped with machine learning algorithms can optimize operational conditions dynamically to maximize efficiency and durability under fluctuating renewable energy inputs.

Operating condition optimization is vital to unlocking higher performance and extended lifetimes for PEMWE systems. Research should focus on understanding and mitigating the effects of high- and differential-pressure operating modes, which often lead to mass transport limitations and membrane degradation. Advanced diagnostic tools and simulation models can provide insights into these phenomena, enabling the development of components and operational protocols that ensure long-term stability under challenging conditions.

Finally, achieving economic and environmental sustainability requires a holistic approach. Comprehensive life cycle assessments (LCAs) should be conducted to quantify the environmental impact of PEMWE systems across their entire lifecycle, from raw material extraction to end-of-life recycling. These assessments can identify opportunities to minimize the carbon footprint and improve resource efficiency. Cost-reduction pathways, such as incorporating recycled or renewable materials and employing energy-efficient manufacturing processes, should also be prioritized. Furthermore, integrating PEMWEs into hybrid energy systems, where excess renewable energy can be stored as hydrogen, could enhance the economic feasibility and operational flexibility of these systems.

In conclusion, the future of PEMWE technology depends on a concerted effort to address the current limitations through innovative research and development. By focusing on materials, manufacturing, system integration, and sustainability, PEMWE systems can transition from pilot-scale applications to global-scale solutions, playing a pivotal role in achieving a carbon-neutral energy economy.

## 8 Results and Discussion

In this review, the component materials of PEMWE systems and the development stages of PEMWE stack systems are examined in detail. In addition, the production and material technology status of the PEMWE cell and its compositions, as well as the commercial status and applications of PEMWEs, are discussed. In the literature, most current studies have been performed to reduce the production costs of PEMWE systems and maintain operating stability. This state reveals the difficulties faced by PEMWE systems. These problems must be solved for these systems to become widespread. The development of materials for PEMWEs is an inevitable result of advancing technology because the parts in PEMWE can be physically and chemically improved by coating both composite structures and materials. In addition, it is judged that these material-based technological developments will contribute to PEMWE and other electrochemical conversion systems in the short and long-term. Many researchers are working on making innovative production techniques suitable for mass production. The 3D printing method, called AM, is seen as a cost-effective production method among these production methods because it has a prominent place in producing complex structures that cannot be produced with traditional manufacturing methods. Complex structures can be produced quickly and relatively cheaply by using the AM method. With the development of AM technology, PEMWE systems will also benefit from these developments. When considering BP and GDL equipment, carbon-based materials are proven elements for the cathode side of the PEMWEs. However, the search for materials with high corrosion resistance on the anode side continues. SS materials instead of Ti are predicted to increase in the future. In addition, the efficiency of PEMWE systems with different innovative composite coating materials to be applied to this equipment. Although the ionic permeability of the membrane is the most critical parameter, it needs to adapt to variable parameters, such as temperature, current density, pressure, and humidity. Nafion membranes have recently been widely used in PEMWE applications thanks to their advantages such as different thicknesses, flexibility, and suitability for production. In the literature, composite catalysts started to take place as PEMWE catalyst coating materials, apart from pure oxides. PGMs, which are rare in nature, combined with lower-cost materials for improving PEMWE performance, are important in reducing costs and conserving scarce resources. It is also thought to impact the development of synthesis methods and material technologies. In studies in the literature, it has been demonstrated that the amount of catalyst loading has been reduced daily, and this situation negatively affects the total cost. PEMWEs are classified at three different pressures, according to their working pressure. PEMWEs under low pressure have a design that can withstand up to 20 bar of pressure, while medium pressure PEMWEs can withstand pressure up to 40 bar. PEMWEs have started to be used as a stack in the laboratory environment and in commercial applications. Although these commercial products have resulted in project-based prototype work for many companies, PEWWEs are available in the market that are mass-produced and can operate at 30 bar. However, when the literature studies are examined, it is stated that a PEMWE can only operate at 30–40 bar outlet pressure, and high-pressure PEMWEs have an operating pressure from 40 to 700 bar. The development of high-pressure PEMWE up to 700 bar is limited due to a lack of technology. Research and development continues to improve high-pressure PEMWE stack components. As a result, it is estimated that PEMWE systems will be used more widely, especially in the development of materials and production techniques. Thus, it can be concluded that high-pressure PEMWEs will be important in future studies. Industries believe that the low-cost, high-volume-specific energy density for PEMWE is a potential choice. Therefore, it is of immense importance for commercial applications of PEMWEs and has gradually become a research point.

Moreover, optimizing the stability and conductivity properties of the components in the stack is of great importance to improve the performance of PEMWE stacks. The design and manufacturing techniques of GDLs have a direct effect on the overall efficiency of the electrolyzer, and improving these components can contribute to improved stability and conductivity. The optimization of material selection and coating methods at the interface between the electrode and the membrane can enhance the long-term durability and performance of the system by increasing ion and electron transport. The use of innovative manufacturing techniques, such as coating methods and 3D printing technology, could allow for more precise control of the microstructure of electrodes. This state can improve the electrical conductivity and electrochemical stability by increasing the homogeneity of the electrode surfaces. Future studies can be achieved to improve the performance of PEMWE stacks through the optimization of the electrode coating thickness, material components, and geometric arrangements. Such innovative improvements could increase the efficiency of PEMWEs while also ensuring their long-term reliability, thereby making them more suitable for commercial use.

## List of Abbreviations

3D: Three-dimensional
AM: Additive manufacturing
AWE: Alkaline water electrolysis
BP: Bipolar plate
CAD: Computer-aided design
CAM: Computer-aided manufacturing
CVD: Chemical vapor deposition
DOE: United States Department of Energy
FDM: Fused deposition modeling
GDL: Gas diffusion layers
HER: Hydrogen evolution reaction
ICR: Interface contact resistance
MEA: Membrane electrode assembly
NREL: National Renewable Energy Laboratory
OER: Oxygen evolution reaction
PEM: Polymer electrolyte membrane
PEMWE: PEM water electrolyzer
PFSA: Perfluoro sulfonic acid
PGM: Platinum group metals
rGO: Reduced graphene oxide
RP: Rapid prototyping
SLA: Stereolithography
SLS: Selective laser sintering
SOEC: Solid oxide electrolysis
SPE: Solid polymer electrolyte
SS: Stainless steel
